# ISOWN: accurate somatic mutation identification in the absence of normal tissue controls

**DOI:** 10.1186/s13073-017-0446-9

**Published:** 2017-06-29

**Authors:** Irina Kalatskaya, Quang M. Trinh, Melanie Spears, John D. McPherson, John M. S. Bartlett, Lincoln Stein

**Affiliations:** 1 Informatics and Bio-computing, Ontario Institute for Cancer Research, Toronto, Ontario Canada; 2 Transformative Pathology, Ontario Institute for Cancer Research, Toronto, Ontario Canada; 3 Department of Laboratory Medicine and Pathobiology, University of Toronto, Toronto, ON Canada; 4 Edinburgh Cancer Research UK Centre, MRC IGMM, University of Edinburgh, Edinburgh, UK; 5 Department of Molecular Genetics, University of Toronto, Toronto, Ontario Canada; 6 Department of Biochemistry and Molecular Medicine, University of California Davis, Sacramento, California USA

**Keywords:** Next-generation sequencing, Somatic mutation, Matching normal tissue, Variant classification

## Abstract

**Background:**

A key step in cancer genome analysis is the identification of somatic mutations in the tumor. This is typically done by comparing the genome of the tumor to the reference genome sequence derived from a normal tissue taken from the same donor. However, there are a variety of common scenarios in which matched normal tissue is not available for comparison.

**Results:**

In this work, we describe an algorithm to distinguish somatic single nucleotide variants (SNVs) in next-generation sequencing data from germline polymorphisms in the absence of normal samples using a machine learning approach. Our algorithm was evaluated using a family of supervised learning classifications across six different cancer types and ~1600 samples, including cell lines, fresh frozen tissues, and formalin-fixed paraffin-embedded tissues; we tested our algorithm with both deep targeted and whole-exome sequencing data. Our algorithm correctly classified between 95 and 98% of somatic mutations with F1-measure ranges from 75.9 to 98.6% depending on the tumor type. We have released the algorithm as a software package called ISOWN (Identification of SOmatic mutations Without matching Normal tissues).

**Conclusions:**

In this work, we describe the development, implementation, and validation of ISOWN, an accurate algorithm for predicting somatic mutations in cancer tissues in the absence of matching normal tissues. ISOWN is available as Open Source under Apache License 2.0 from https://github.com/ikalatskaya/ISOWN.

**Electronic supplementary material:**

The online version of this article (doi:10.1186/s13073-017-0446-9) contains supplementary material, which is available to authorized users.

## Background

Somatic, or acquired, mutations are genetic changes that accumulate in the non-germline cells of an individual during his or her lifetime. Somatic mutations that disrupt genes involved in one or more of the pathways that regulate cell growth, programmed cell death, neovascularization, and other “hallmarks of cancer” can lead to the development of a neoplasm [1–4]. The use of next-generation sequencing to comprehensively characterize cancer genomes has led to multiple breakthroughs in the understanding of driver genes and pathways involved in cancer [5–7], the interaction between environmental exposures and patterns of mutations [8, 9], tumor classifications [10, 11], and the evolution of tumors in the presence and absence of therapy [12, 13].

Accurate identification of somatic mutations is an essential first step for many cancer studies. There are many challenges in mutation calling, including but not limited to: (a) the admixture of multiple tumor subclones with each other and with normal tissue; (b) the frequent presence of copy number alterations in tumors; and (c) a raw error rate from sequencing instruments that is comparable to the variant allele frequency of mutant alleles in admixed samples. Nevertheless, the current generation of somatic mutation calling tools are highly accurate, even in the presence of admixed samples with low variant allele frequencies [14–17]. However, all these tools require both patient’s tumor and normal tissues (typically white blood cells or adjacent normal tissue in the tumor resection specimen) in order to distinguish somatic mutations from uncommon germline polymorphisms. These tools construct a multiple alignment with both the tumor and normal reads, and then scan down the columns of the alignment to identify tumor-specific alterations, using statistical models of sequencing error rates and base quality scores to reduce false positives.

In some commonly encountered scenarios, however, matching normal tissues are not available. This may be because normal samples were not collected in the first place, or because the patient consent was obtained in a way that precludes examination of normal tissue or germline variants. This is most commonly encountered when performing analysis on retrospective studies with human material from clinical trials, pathology archives, and legacy biobanks, a strategy that may be required when building a cohort of a rare cancer type or subtype, or when executing secondary studies on clinical trials. Another common scenario is the use of a cancer cell line as an experimental model, many of which have no information on the donor’s normal genomes. There may also be financial considerations; sequencing both tumor and normal genomes not only roughly doubles the cost but also increases data storage and computational requirements. In these cases, there is a need to identify somatic mutations from tumor tissues without the presence of the normal tissues.

One of the main challenges for accurate identification of somatic mutations in the absence of normal DNA is to distinguish somatic mutations from germline polymorphisms (single nucleotide polymorphisms (SNPs)). On average, the genome of any human individual contains ~3,300,000 SNPs [18]. Roughly 20,000–25,000 of those are coding variants and 9000–11,000 are nonsynonymous [19]. All common SNPs with population frequencies of 1% or greater in the major world population groups have been extensively catalogued [20], and these can be excluded from consideration by a simple filtering step. Some ethnic subpopulations are under-represented and appropriate calibration within these groups may be required. In addition, however, each individual is estimated to carry 400,000–600,000 rare SNPs specific to the individual or his or her close family [19], and these cannot easily be excluded by comparison with SNP databases or with recent large-scale exome sequencing projects.

In this study, we describe an algorithm that uses supervised machine learning to distinguish simple substitution somatic mutations in coding regions from germline variants in the absence of matching normal DNA. The accuracy of this approach, calculated based on the whole-exome sequencing data from The Cancer Genome Atlas (TCGA), as well as targeted (gene-panel) sequencing performed on formalin-fixed paraffin-embedded (FFPE) tissue, lies in a range that would be acceptable for most applications.

## Implementation

### Validation sets

Protected datasets in VCF format (containing both somatic and germline variants) were downloaded directly from TCGA portal. Only one sample (TCGA-IB-7651-01A from PAAD) was excluded from the analysis based on its extremely high mutational loads (~300-fold in comparison to the median for this cancer set). According to the headers of the retrieved VCF files, variant calling in KIRC (kidney renal clear cell carcinoma), PAAD (pancreatic adenocarcinoma), and COAD (colon adenocarcinoma) sets was done using the Baylor College of Medicine (BCM) CARNAC (Consensus And Repeatable Novel Alterations in Cancer) pipeline (version 1.0) [21]; in BRCA (breast invasive carcinoma) and UCEC (uterine corpus endometrial carcinoma) sets with the bambam pipeline (version 1.4) from University of California at Santa Cruz (UCSC; Sanborn JZ, Haussler D; University of California; Bambam: parallel comparative analysis of high-throughput sequencing data. Patent. EP2577538 A1). During quality control of the validation sets, we noticed that, of the five TCGA datasets used for validation, the KIRC, PAAD, and COAD sets did not contain any homozygous variants, possibly a consequence of CARNAC filtering. To maintain consistency across all five data sets, we removed all homozygous variants from UCEC and BRCA as well.

In addition, we downloaded 145 ESO (esophageal adenocarcinoma) BAM files from dbGAP portal (https://www.ncbi.nlm.nih.gov/projects/gap/cgi-bin/study.cgi?study_id=phs000598.v2.p2 [22]). We extracted the raw reads from the BAM files and aligned them to human genome hg19 using BWA (v0.6.2) [23]. Collapsed reads that aligned in the correct orientation were passed to Mutect2 (bundled with GATK v3.6) [17] to call variants. MuTect2 was run twice on each sample in two different modes: (1) in the usual mode with pair matching normal to retrieve gold-standard somatic mutation calls; and (2) in so called tumor_only_mode to call all variants (including all somatic and some germlines). This mode mimics the situation when matching normal data are not available. Variants from 100 ESO samples were randomly selected and used for training set generation and the remaining samples for validation.

ANNOVAR (version released on 2012-03-08) was used for coding region functional annotations [24]. Variants were filtered based on the following criteria: (1) minimum coverage of at least 10×; (2) PASS filtering; (3) exclusion of all non-single nucleotide variants (non-SNVs; e.g., indels or multiple base substitutions); (4) removing all variants with “N” as reference alleles; and (5) exclusion of all variants that were labeled as “unknown” by ANNOVAR. The basic statistics of each dataset are shown in Table 1. The use of TCGA and ESO data sets was authorized under dbGaP project #6257.

**Table 1:** Characteristics of cancer datasets used for training and/or validation

Dataset (source)	Number of samples	Number of samples used in testing	Mean read depth after filtering [95% CI]	Mutation calling pipeline	Total number of somatic/germline SNVs^a^ in all samples	Mean somatic SNVs per sample [95% CI]^a^	Mean germline per sample [95% CI]^a^	Ratio somatic to germline (after collapsing)
UCEC (TGCA)	251	151	88.84 [88.77, 88.92]	bambam_v1.4	38,012/504,241	147.015 [88.36, 205.66]	2,008.92 [1,972.23, 2,045.62]	2:1
BRCA (TCGA)	500	400	85.92 [85.87, 85.97]	bambam_v1.4	5556/1,037,432	10.77 [9.05, 12.48]	2,074.86 [2051.26, 2098.46]	1:6
COAD (TGCA)	215	115	122.17 [122.06, 122.28]	carnac_v1.0	60,624/1,932,510	276.68 [191.78, 361.58]	8,988.41 [8826.01, 9150.82]	1:1
KIRC (TGCA)	304	204	177.59 [177.46, 177.73]	carnac_v1.0	10,489/2,416,155	33.56 [31.60, 35.51]	7,947.87 [7792.68, 8,103.07]	1:7
PAAD (TCGA)	146	46	363.09 [362.80, 363.37]	carnac_v1.0	5,593/1,263,918	37.08 [33.59, 40.58]	8,656.48 [8587.71, 8725.25]	1:10.5
ESO (dbGAP)	145	45	58.39 [58.33, 58.44]	MuTect2	26,098/790,051	181.85 [150.65, 213.05]	5,451.51 [5,307.16, 5595.85]	1:2.5

All datasets were sequenced using Illumina technology

^a^Only non-silent variants in coding regions with read depth >10 and PASS somatic mutation caller filtering were taken into account

### Variant annotations

Each variant in every validation cancer set was annotated using COSMIC v69 [25], dbSNP v142 [20], Mutation Assessor [26], ExAC r0.3 [27], and PolyPhen-2 [28]. Annotation against the dbSNP database produced two outputs: (1) whether a variant was catalogued by the “common_all” division of dbSNP (found in ≥1% of the human population by definition); or (2) represents a rare polymorphism. COSMIC v69 was released prior to the availability of TCGA or ESO data sets used for validation, and is therefore not contaminated with somatic mutations from those sets. (The first COSMIC release to contain data from any of these sets was version 72). Future users of ISOWN are encouraged to use the latest version of COSMIC.

### Supervised learning

WEKA (Waikato Environment for Knowledge Analysis) software v3.6.12 suite [29], a mature Java-based machine learning toolkit, was employed for the variant classification task. The WEKA toolkit provided a collection of machine learning algorithms for data mining together with graphical user interfaces. Algorithms used in the study are described in Additional file 1: Supplemental methods.

The performance of all classifiers was evaluated by tenfold cross-validation, and the following six measures were used to estimate classifier performances:

1. *Recall* (or sensitivity or true positive rate) measures the proportion of the known somatic variants that are correctly predicted as those and is defined as *TP*/(*TP* + *FN*), where TP is true positive and FN is false negative.
2. *Precision* is a fraction of the correctly called somatic mutations to all variants that are labeled as somatic by the classifier and is defined as *TP*/(*TP* + *FP*), where FP is false positive.
3. *F1*-*measure* [30] is the harmonic mean of precision and recall: 2 × (Precision × Recall)/(Precision + Recall).
4. *False positive rate* (FPR) is the fraction of germline variants incorrectly classified as somatic and is defined as *FP*/(*FP* + *TN*), where TN is true negative.
5. *Accuracy* (ACC) is the proportion of variants that are correctly predicted and is defined as (*TP* + *TN*)/(*TP* + *FN* + *TN* + *FP*).
6. *Area under ROC curve* (AUC) denotes the probability that a classifier assigns a higher score to the positive instance than a randomly chosen negative sample. It measures the general ability of the classifier to separate the positive and negative classes. The best performing classifier for each cancer dataset was selected based on AUC and F1-measure.

### External and internal features

All features used for variant classification are shown in Table 2. Variants are described by ten features that ultimately contributed to subsequent machine learning training and evaluation steps. One class of features came from external databases, and the other class was derived from the characteristics of the variants themselves.

**Table 2:** List of features used in the classifiers, types of their values, and source of data

Features	Type of value	Internal or external	Number of distinct values
COSMIC_CNT	Integer	External database	Numeric
ExAC	Boolean	External database	2
dbSNP	Boolean	External database	2
Mutation assessor	Categorical	External database	5
PolyPhen-2	Categorical	External database	3
Sequence context	Categorical	Human genome	64
Sample frequency (SF)	Double	Internal data	Numeric
Variant allele frequency	Double	Internal data	Numeric
Flanking regions	Double	Internal data	Numeric
Substitution pattern	Categorical	Internal data	6

Features based on external databases:

1. The Catalogue Of Somatic Mutations In Cancer (COSMIC) [25] is by far the richest database of the cancer-related somatic mutations. The presence of a candidate variant in COSMIC is predictive, but not definitive, of a somatic origin. The biggest drawback of COSMIC (v69) usage is that more than 90% of all coding somatic SNVs catalogued by COSMIC were submitted from a single sample. Most of these are random passenger mutations. In practice, therefore, we used the COSMIC CNT (instead of just acknowledging the presence of a variant in this database) attribute as the feature presented to machine learning. CNT is an attribute assigned to each coding variant catalogued by COSMIC and represents a number of samples with a mutation across all tumor types. The CNT value was used as a feature in the classifier. If the variant wasn’t catalogued by COSMIC, this value of the numeric feature was assigned to zero. Thus, CNT varies from 0 to 19,966 (a well-described mutation in BRAF).
2. Correspondingly, the Exome Aggregation Consortium (ExAC) has collected germline variants from ~60,000 independent individuals and is one of the richest databases of common germline polymorphisms. A boolean feature based on the presence in ExAc (is.in.ExAc/not.in.ExAc) was assigned to each variant in our validation sets and used as an independent feature.
3. The dbSNP resource, another widely used collection of the common germline variants, classifies submitted variants into common (≥1% of the human population) and rare polymorphisms. All variants in validation sets were annotated against dbSNP/common_all and dbSNP/rare databases. The information from the first set was used for variant pre-labeling (see the “Variant pre-labeling” section) and therefore was not used again for the classifier. The second annotation was used as an independent feature in the classifier.
4. *Sequence context* is defined as the three-base sequence comprising the variant and its flanking bases. It is known that different cancer types have different mutational signatures [31]. In addition, sequence context can help to distinguish germline from somatic mutations due to the differences in the mutational processes that often, but not always, generate these two types of change. For example, we have noticed that in all six cancer sets somatic mutations are significantly enriched in the AGA pattern and germline polymorphisms in the ATG pattern.
5. Mutation Assessor predicts the functional impact of amino acid substitutions in proteins based on evolutionary conservation of the affected amino acid in protein homologs. We assume that, on average, the impact of the somatic mutation on protein function will be significantly higher than a germline polymorphism. Categorical output from Mutation Assessor (high, medium, low, or neutral) was used as a feature in the classifier. Stop loss and especially stop gain mutations (annotated by ANNOVAR) usually have greater impact on protein function and predominantly occur as somatic alterations. As variants that introduce stop gain or stop loss are ignored by Mutation Assessor and mutually exclusive to its output; these mutation types were added as categories of the feature.
6. PolyPhen-2 is a tool that predicts damaging effects of missense mutations based on both sequence and structural information. It was also used as an independent feature in the classifier.

With respect to the use of functional impact features, while a small number of germline polymorphisms may have high protein structure impact, we confirmed that in all sets used for validations, somatic mutations are significantly enriched in “high” and “medium” impacts, whereas germline polymorphism are enriched in “neutral” impacts. For example, the ratio of germline polymorphisms scored as neutral impact by Mutation Assessor ranged from 40 to 45% depending on cancer data set, while neutral somatic mutations occurred 23–27% of the time (Additional file 1: Table S6). A similar difference was observed for PolyPhen-2 output (Additional file 1: Table S7).

The following four features are generated based on internal characteristics of the variants themselves: s*ample frequency*, *variant allele frequency*, *substitution pattern*, and *flanking regions* (Table 2).

Internal annotations:

7. *Sample frequency* is calculated as the fraction of samples carrying that particular variant over the total number of samples in the particular dataset. Variants with high sample frequencies are more likely to be germline polymorphisms. More detailed justification of this feature is provided in the Additional file 2: Figure S4.
8. *Variant allele frequency* (VAF) is calculated as the ratio of number of reads supporting the variant allele over the total number of reads. The heterozygous VAF distribution is centered at 50% [32] for germline polymorphisms; however, germline VAFs can deviate from 50% when they are involved in a somatic copy number alteration event. VAFs for somatic mutations are more likely to have values below 50% due to copy number variation, admixture with normal tissues and/or tumor subclonality, and, on average, range from 22% to 50% [7] and in some cases reach values greater than 50% due to amplification events (Additional file 2: Figure S3).
9. *Flanking regions*: The VAF of each variant is an informative feature due to the fact that somatic mutations tend to be subclonal, while heterozygous SNPs will have a VAF close to 50%. To use VAF as a predictive feature, we examine regional differences in VAF between the candidate variant and flanking polymorphisms. For each candidate variant (X) we searched for flanking polymorphisms (that were catalogued by dbSNP/common) within 2 Mbp of flanking 5′ or 3′ regions from X (Additional file 2: Figure S1a). The 5′ and 3′ flanking region polymorphisms are labeled as V1 and V2, respectively. If both V1 and V2 exist and the 95% confidence intervals (CIs) of their VAFs, as determined by the binomial distribution, overlap the 95% CI of X, then X is more likely a germline variant. On the other hand, if the VAF CI for X overlaps the CI for neither V1 nor V2, while the V1 and V2 CIs overlap with each other, then X is most likely a somatic variant. In all other cases, including where V1 and/or V2 were not found within the 2-Mbp flanking regions, this feature is marked as NA (not applicable). The flanking region feature measures whether the VAF of an unknown variant is similar to the VAF of flanking known germline polymorphisms. Because copy number alterations are often quite large, germline polymorphisms are expected to have similar VAFs to those of flanking SNPs, while a somatic mutation VAF should be different from its flanking SNPs. This feature strongly depends on the presence of known germline polymorphisms in close proximity to an unclassified variant, and because of this and the strict conditions for defining informative flanking SNPs, this feature is unavailable for up to 50% of the variants in a typical cancer exome.
10. *Substitution pattern* is defined as a two base sequence that contains the reference (wild type) and the newly introduced variant base of the mutation. For example, the substitution pattern of chr3,178936094C > G mutation is “CG”. All substitution patterns are combined into six categorical subtypes: “CA”, “CG”, “CT, “TA”, “TC”, and “TG”. We determined that somatic mutations (as well as germline polymorphisms) are often enriched in the particular substitution pattern. For example, across all tested datasets somatic mutations were significantly enriched in C > A/G > T substitutions and germline variants were significantly enriched in T > C/A > G exchanges.

### Feature selection

We used the WEKA-InfoGain feature selection tool to ensure all features we selected are relevant and not redundant [33].

### Variant collapsing

For the somatic/germline classification task, we assumed that variants that share the same genomic position and substitution pattern are either somatic or germline across all samples within a particular cancer data set (Additional file 2: Figure S2). We distinguished between the set of *unique variants*, defined as the unique union of all variants (genomic positions + substitution patterns) in the data sets, from the set of *total variants*, which includes all variants across all samples. This simplifies the classification problem: instead of making predictions on a large number of variants (ranges in million; see column 6 in Table 1), we only need to do predictions on a few hundreds of thousands unique variants (Additional file 1: Table S5). Justification of this step is provided in Additional file 1: Supplemental methods (Additional file 1: Table S5). Variant collapsing is the process of transforming the set of *total variants* into the set of *unique variants*.

### Adapting internal machine learning features to the mono-labeled approach

After variant collapsing, the features generated based on external annotations will be identical for all samples in which this variant was found. For example, chr7,140453136A > T in COAD detected in 27 out of 215 samples will have identical values for CNT, ExAC, dbSNP, Mutational Assessor, PolyPhen, and sequence context annotations across all 27 samples. However, as a consequence of variant collapsing, VAF and flanking region annotations might be different for the same variant from sample to sample. Thus, if a variant was called in one sample, its actual VAF value was used in the classifier; otherwise, if a variant was called across two or more samples, the mean of VAFs of all variants is used.

Flanking region assessment was calculated for each variant as either “*true*”, “*false*”, or “*NA*” (described above). If a variant was called in only one sample, flanking region assessment equals “true” was converted into a flanking region feature equals “1” and “false” to “0”. Multiple ambiguous decisions for the same variant across multiple samples were collapsed in the following way: a weight ranging from 0 to 1 for each collapsed variant is calculated as the ratio of “true” counts over the total number of samples with this variant (Additional file 2: Figure S1b). If flanking regions across all samples were all NAs, then the weight is NA.

### Supervised learning algorithms

The full list of the tested supervised learning algorithms together with their short descriptions as well as settings and optimization strategies can be found in Additional file 1: Supplemental methods. In summary, seven algorithms were tested: JRip [34], J48 [35], random forest [36], LADTree [37], naïve Bayes classifier (NBC) [38], logistic regression [39], and support vector machine (SVM) [40].

### Variant pre-labeling

**Fig. 1:**
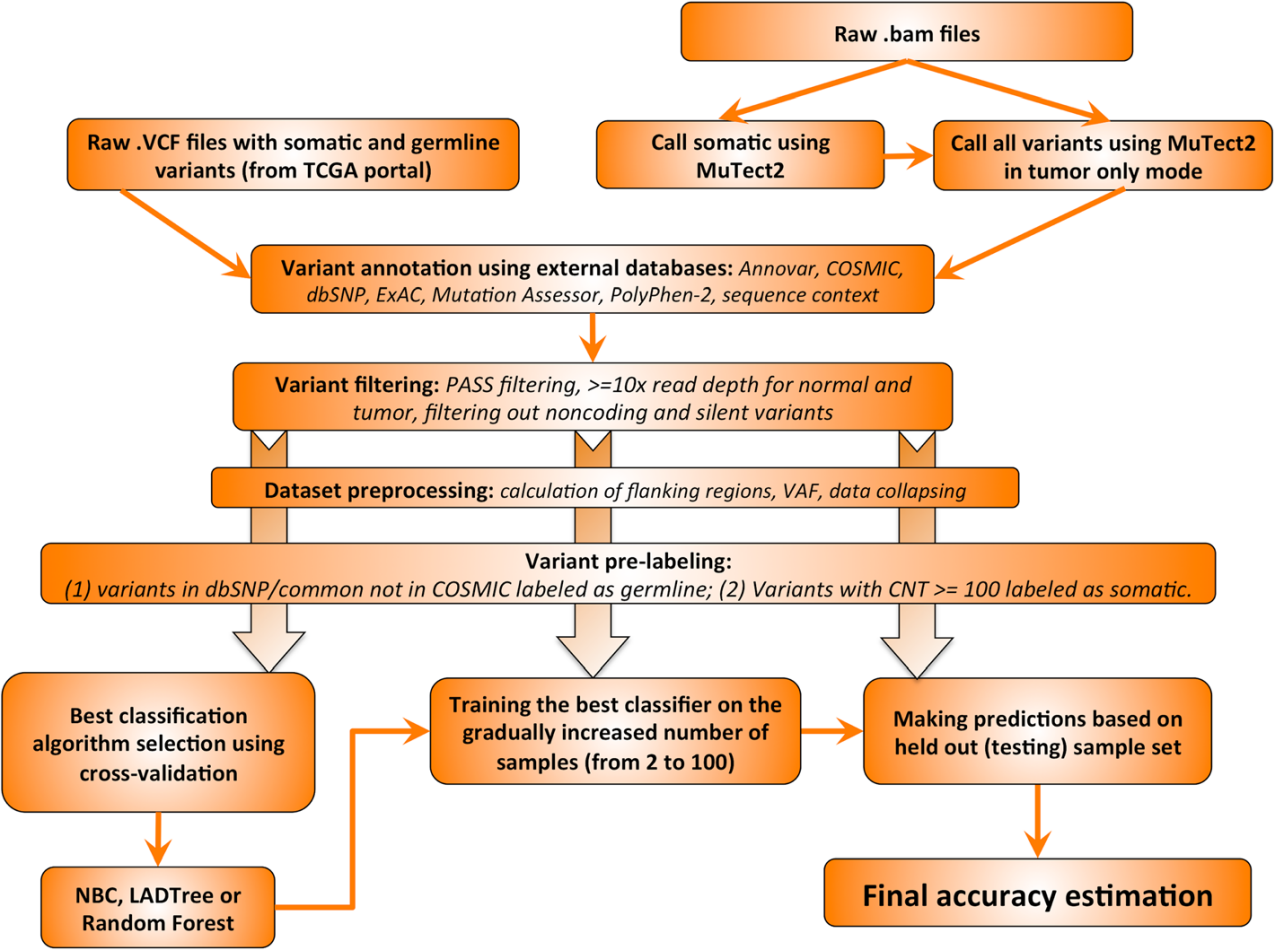
ISOWN framework for somatic mutation prediction. Variants retrieved either directly from TCGA portal in the form of VCF files or using GATK/MuTect2 pipeline (see “Implementation” section for more details) were annotated with a series of external databases. Low quality calls were removed by applying a standard set of filters. Only coding and non-silent variants were taken into account (unless otherwise indicated). After flanking regions and variant allele frequencies were calculated for each variant and data collapsed in the unique set of variants (see “Implementation” section), some variants were pre-labeled as germline based on their presence in dbSNP/common_all but not in COSMIC or as somatic based on the fact that over hundred samples with this particular mutation were submitted to COSMIC (CNT >100). The best machine learning algorithm was selected using a tenfold cross-validation approach. One hundred randomly selected samples from each dataset were used for classifier training and final accuracies were calculated based on the remaining samples

Some subsets of variants do not require classification. For example, the variants that are in dbSNP/common_all and not in COSMIC are most likely germline in origin and were pre-labeled as such; justifications are provided in Additional file 1: Table S3. High values for COSMIC CNT is a good indicator that variants are true somatic mutations (Additional file 1: Table S4), and all variants with CNT ≥100 were pre-labeled as somatic. Pre-labeled variants were not subjected to the classification step (Fig. 1).

### Tenfold cross-validation

Tenfold cross-validation was used to perform the primary assessment of the algorithm performance and to choose the best classification strategy. We generated 1000 training subsets each containing 700 randomly selected somatic mutations and 700 randomly selected polymorphisms for each cancer type. The best classification algorithm was chosen using tenfold cross-validation based on the highest AUC.

### Validation on independent sets

The best classification algorithm chosen during tenfold cross-validation was trained using a linearly increasingly number of samples from 1 to 100 for each cancer set. The validation was done using a separate validation dataset (not used in training) based on: (1) only non-silent variants; (2) only silent variants; (3) somatic mutations occupying different VAF tiers. We also performed cross-cancer validation by training in one cancer type and validating in a different cancer type. The algorithm was also evaluated on an independent pancreatic cancer dataset and a series of cell lines.

## Results

### Development of a somatic prediction pipeline

In this work we focused on predicting single-base substitution somatic mutations in coding regions. Figure 1 illustrates the overall architecture of our prediction algorithm. The design of our pipeline can be summarized as follows: VCF files containing both somatic and germline variants from five cancer types were downloaded from TCGA portal. Only those variants that passed a somatic mutation caller filter (marked with “PASS” in VCF files) with read depth at least 10× were used in the prediction pipeline. Each variant was annotated against ANNOVAR, dbSNP, ExAC, COSMIC, Mutation Assessor, and PolyPhen. Based on functional annotations from ANNOVAR, we removed all non-coding variants as well as variants with unknown annotations.

We chose validation data sets that represent a range of somatic mutation loads and mutation-calling pipelines. For the five validation datasets from TCGA, we used the published somatic mutations and germline polymoprhisms, which were in turn derived from paired tumor–normal samples processed by either the CARNAC or the bambam pipelines (Table 1). In addition, we generated validation data for a sixth data set (145 esophageal adenocarcinoma (ESO) samples) using the popular Mutect2 paired mutation caller [17], starting with unaligned BAM files. Mutect2 was first ran in paired mode on tumor and matched normal to generate the gold standard list of somatic mutations. We then ran Mutect2 in tumor-only mode on the tumor sample only to generate somatic mutations together with germline variants to present to the classifier. The second mode completely mimics the situation when matching normal tissues are not available.

To validate different supervised learning algorithms provided by WEKA, for each tumor type we generated 1000 training sets in Attribute-Relation File Format (ARFF), each containing 700 randomly selected somatic mutations and 700 randomly selected germline polymorphisms. The performance of the machine learning classifiers was evaluated using tenfold cross-validation based on the training sets. This was repeated using classifiers representative of each of the major classification methods (see “List of tested learning algorithms” in Additional file 1: Supplemental materials). The best classification method was chosen based on the highest AUC.

For validation purposes, the sample set was then randomly divided into a training sample subset (100 samples) and a held-out validation sample subset (the remaining samples). Each of the six cancer type data sets was preprocessed and collapsed independently. Using the best classification methods (NBC and LADTree), the classifier was trained with a gradually increasing number of samples from the training set and the accuracy was calculated using the held-out validation sample set.

### Datasets

Evaluation of the classifiers was performed on six different cancer datasets: UCEC (uterine corpus endometrial carcinoma), KIRC (kidney renal clear cell carcinoma), COAD (colon adenocarcinoma), BRCA (breast invasive carcinoma), ESO (esophageal adenocarcinoma), and PAAD (pancreatic adenocarcinoma).

In total, six different tumor types were used for ISOWN validation. All datasets were sequenced using Illumina technology. Average read depth ranged from 58× to 363× (Table 1). The number of samples in each dataset as well as the number of the coding non-silent variants per data set are provided in Table 1. The average number of somatic non-silent mutations in the coding regions per sample ranged across an order of magnitude from 10.77 for BRCA to 276.68 in COAD (Table 1).

Because of the range in somatic mutation and germline polymorphism rate, each of the testing sets contained different ratios of positive (somatic mutation) and negative (germline polymorphism) instances, which allowed us to validate the algorithm in several different settings (Table 1, last column). The ratio of somatic to germline variants ranged from 2:1 in the UCEC set to 1:10.5 in the PAAD set and, surprisingly, did not always correlate with mutational load. For example, BRCA has the lowest mutational load (~10 somatic SNVs per sample; Table 1) but the number of germline variants is only six times higher than somatic variants (in the collapsed set), whereas PAAD has 37 somatic SNVs per sample but the ratio of somatic to germline variants reaches 1:10. It is unlikely that the rate of germline SNPs varies to this extent across TCGA cancer cohorts, and most likely these differences reflect disparities in the approaches used to call and filter variants in these datasets. Our algorithm was nevertheless able to learn and correctly discriminate somatic from germline variants across a wide range of absolute variation counts and somatic to germline ratios.

### Tenfold cross-validation and the best classification method selection

We first set out to select the best classifier(s) for each cancer dataset, investigate whether the best classifier is cancer-specific, and to compare performance measures across different cancer types. We present the results from the best trained models for only the seven supervised learning algorithms we selected, although several others were investigated (Additional file 1: Supplemental methods).

The performance measures presented here were retrieved based on collapsed datasets (see the “Variant collapsing” section) without taking into account pre-labeled variants. Cross-validation was done based on 1000 training sets, each balanced with 700 somatic and 700 germline variants randomly selected from each cancer set (Fig. 1 and “Implementation” section).

**Fig. 2:**
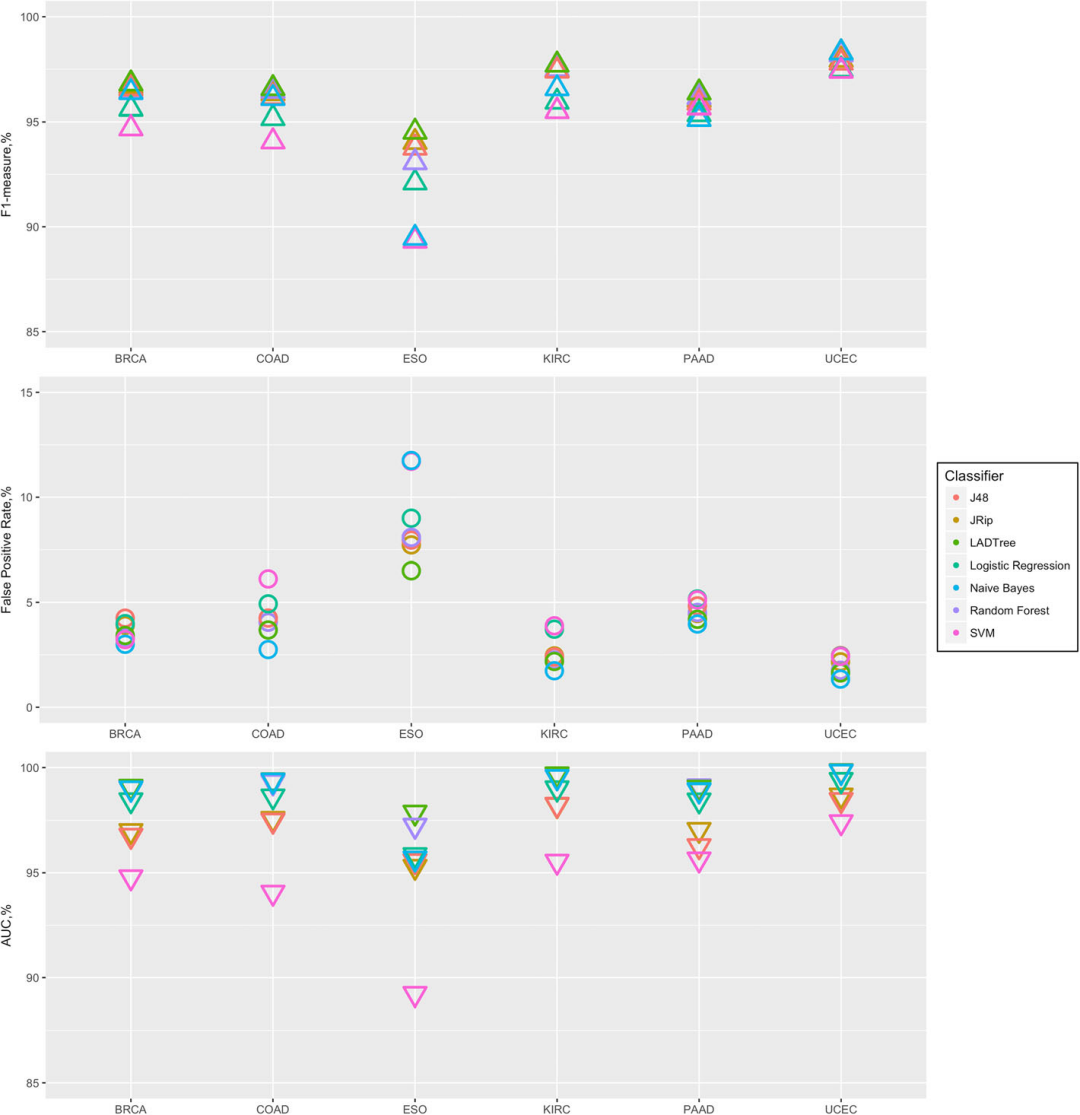
Tenfold cross-validation. We generated 1000 training sets, each containing 700 randomly selected somatic and 700 germline variants from each cancer set. ISOWN validation was done using different machine learners (shown with different colors). Plot shows average F1-measure (*upper panel*), false positive rate (*middle panel*) and AUC (*lower panel*) from 1000 training sets

Figure 2 shows performance measures from tenfold cross-validation for all cancer datasets. The top panel shows similar performances for five out of six cancer datasets regardless of which supervised learning method was used. ESO is the only dataset with slightly lower F1-measure (ranges from 88 to 95%). Overall, all seven selected classifiers showed comparable performances in each of the six cancer data sets we tested, ranging from ~3–4%.

The false positive rate (FPR) was less than 7% for all datasets except ESO. Usage of NBC consistently shows the lowest FPR below 5% for all but the ESO set. The FPR for the ESO set ranges from 6 to 12% (Fig. 2, middle panel).

Based on the AUC, the worst classifier in all six cases was SVM in spite of the fact that both kernels, Poly-kernel and RBF-kernel, were tested and optimized. The AUC for the best classifiers was estimated to be ~99% for COAD, UCEC, KIRC, and BRCA, ~98% for PAAD, and ~96% for ESO (Additional file 1: Table S1). Based on mean AUC value, NBC and LADTree were chosen as the best classification algorithms (Fig. 2, bottom panel) for all cancer sets but ESO. Random forest and LADTree were used for ESO.

### Classifier validation and effect of training set size on performance

The final assessment of the classifier performance was done based on the held-out validation testing sample sets that had not been used in the training procedure (see pipeline description and Fig. 1). In addition, we investigated the effect of the size of the training set on the final performance measures. The validation was performed as follows: the indicated classifier was trained based on gradually increasing number of samples (starting from 2 to 100 with increments of one) and for each case, accuracy, F1-measure, and FPR were calculated based on the held-out testing set. The training set was generated based on all somatic variants retrieved from the indicated number of samples plus an equal number of randomly selected germlines.

**Fig. 3:**
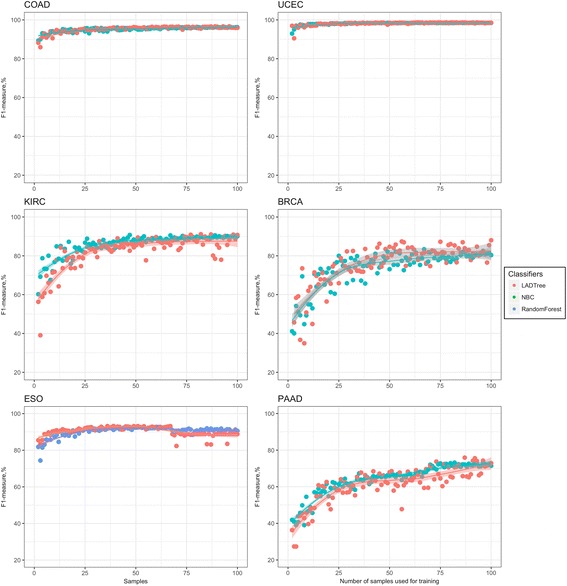
ISOWN validation using different machine learning algorithms for six whole-exome sequencing datasets. NBC (*green*), LADTree (*red*), and random forest (*blue*) were trained based on a gradually increasing number of samples (*x-axis*). The F1-measure was calculated based on a held-out independent sample set across six cancer datasets

The overall accuracies for all six cancer sets is over 99.0% for almost all training sets (Additional file 3: Table S2). But the FPR and F1-measure are better measurements of a classifier’s performance when the data set is unbalanced, as it is in the validation sets used in this study. The FPR was below 0.5% if the classifier was trained with at least 25 samples for the COAD, UCEC, KIRC, and BRCA sets, and at least 50 samples for PAAD and ESO (Additional file 2: Figure S5). The F1-measure was high (above 90%) in four out of six studied cancer sets and reached 91.1% for KIRC, 93.2% for ESO, 96.6% for COAD, and 98.6% for UCEC. BRCA, with a max F1-measure of 88%, showed slightly reduced but still acceptable performance. PAAD had the worst accuracy, with the F1-measure reaching a maximum of just 76% (Fig. 3).

The comparison of performance between the two best classifiers, LADTree and NBC (random forest for ESO), is depicted in Fig. 3 and Additional file 2: Figure S5. When applied to the BRCA, COAD, and UCEC tumor types, NBC and LADTree classifiers were indistinguishable. In KIRC and PAAD, NBC significantly outperformed LADTree in terms of accuracy. LADTree and random forest showed no differences in performance in ESO.

The F1-measure plateaus for all cancer sets but PAAD, most likely due to low mutation load. Thus, we recommend using at least 25 samples for training of highly mutated cancer types (like COAD, ESO, and UCEC) and 50–100 samples for medium mutated types (like BRCA and KIRC) and >100 samples for cancers with a low mutation load (like PAAD). Recall and precision for the above described experiments are listed in Additional file 3: Table S2.

### ISOWN performance on silent mutations

Some applications require a list of silent somatic mutations in addition to non-synonymous ones. We evaluated the accuracy of our classifier for distinguishing silent somatic mutations in coding regions. In this scenario, PolyPhen and Mutation Assessor do not provide functional annotations for most variants. Thus, we expected that the performance of the classifier would be slightly lower due to missing functional annotation features.

We performed training and validation in a similar manner as described earlier: training using nonsynonymous variants from increasing number of samples from each cancer set and validating with either non-silent variants only (as it was done in the previous experiment) or silent variants only. As LADTree showed better or comparable performance (see “Classifier validation and effect of training set size on performance” section) in the majority of the datasets, it was selected for this and following experiments. For the purposes of comparison, F1-measures are shown for predictions of both silent and non-silent somatic mutations in Additional file 2: Figure S6. In all six tumor types the F1-measure was reduced for silent mutation prediction versus non-silent. The effect was relatively small for UCEC, ESO, and COAD, with reductions in F1-measure of 1.9, 2.3, and 3.5%, correspondingly. Other tumor types showed a stronger effect: F1 was reduced by 8.9, 11.9, and 17.7% in KIRC, PAAD, and BRCA, respectively, when applied to silent variants (Additional file 2: Figure S6). We also observed that the classifiers plateaued at roughly the same number of training samples regardless of whether silent or non-silent variants were tested (Additional file 2: Figure S6).

In summary, the ISOWN algorithm can correctly classify silent coding variations at acceptable levels in tumor types with high and moderate mutational loads (F1 92–97% for COAD, ESO, and UCEC, 80–87% for BRCA and KIRC), but has error rates that are unacceptably high in tumors with low mutational loads (69.2% for PAAD).

### ISOWN performance in relationship to VAF

Depending on the cellularity and heterogeneity of the tumor sample, the VAF of somatic mutations may vary significantly. Accurate calling of low-VAF mutations is important for identification and characterization of subclones present in the tumor. To address this issue, we studied the impact of VAF on ISOWN accuracy. For this experiment, we trained the LADTree classifier according to the protocol described earlier, but divided the somatic mutations used in the testing sets into two sets based on their collapsed VAF values: low VAF variants (VAF ≤ median of all collapsed somatic variants) and high VAF. To maintain the original ratio of somatic and germline variants in the testing set, germline polymorphisms were randomly divided among the two test sets.

As we expected, ISOWN shows consistently better performance for predicting somatic mutations with low VAF in comparison to high VAF. The median VAF varied from 11.3% in the PAAD set to 31.7% in the UCEC set (Additional file 2: Figure S2). In spite of this wide variation, we observed only minor differences in the F1-measure (in the range of 0.1–2.9% differences) in the majority of tumor types. The most significant differences were observed in ESO, where we observed a reduction of 4.3% in the F1-measure for somatic mutation classification for low versus high VAF test sets (Additional file 2: Figure S7). In conclusion, ISOWN performs well in predicting somatic mutations across differing VAF tiers.

### ISOWN performance on cross-cancer type training and testing

In some cases, it may be difficult to find a sufficient number of samples sequenced with matching normal tissues to train the classifier, especially for rare cancer types. We decided to test ISOWN in a setting in which the classifier was trained using one cancer type and then tested on another cancer type.

**Fig. 4:**
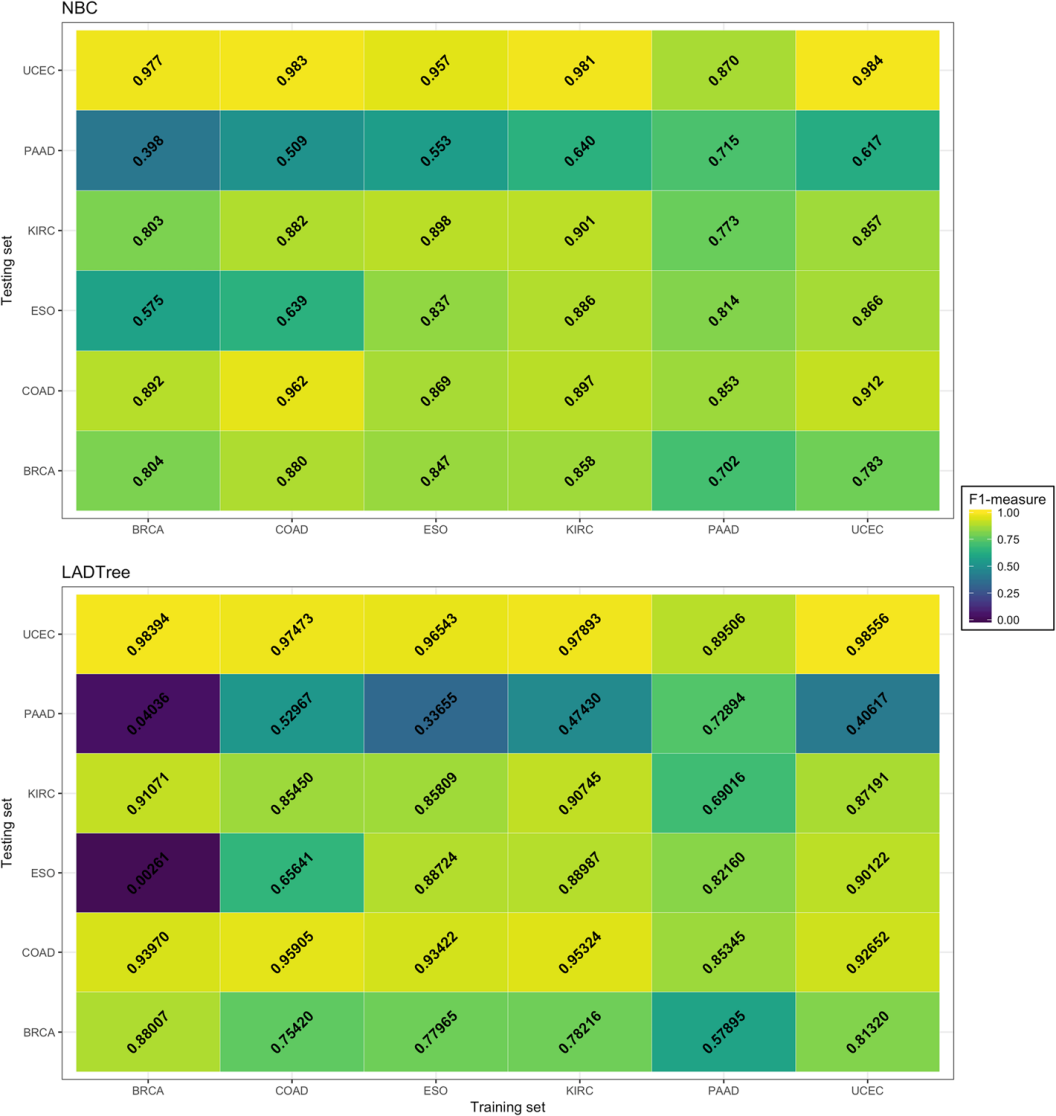
Cross-cancer validation. NBC (*upper panel*) and LADTree (*lower panel*) classifiers were trained using variants from 100 samples from cancer indicated on the *x-axis* and validated using cancer set indicated on the *y-axis*

Figure 4 shows the results from cross-cancer type testing. The first conclusion is that in all six cancer types (with minor exceptions), training and testing using the same cancer type give the best accuracy. This is explained by the following differences between cancer types: (a) VAF distributions; (b) different patterns of sample frequencies; (c) different mutation signatures; and probably (d) different calling biases in among TCGA variant call sets. The second observation is that the somatic mutation prediction in the PAAD set posed the greatest difficulty for the classifier among all six training sets, most likely due its high ratio of germline to somatic mutations.

It is interesting to note that the UCEC and KIRC training sets in combination with NBC demonstrated relatively good accuracy across all six sets; these training sets can probably serve as uniformly applicable training sets for cancers with medium to high mutational loads. The LADTree classifier was consistently worse than NBC in this experiment. In summary, cross-tumor type training can produce accurate classifiers, and in at least one case differences in the paired variant calling methodology are more important than differences between the tumor types.

### Misclassified variants

To understand the source of misclassifications, we examined these variants in greater detail. To do so, we trained the classifier on variants retrieved from 100 samples in each cancer data set and collected all misclassified variants. In the discussion below, germline variants misclassified as somatic by our algorithm are called false positive variants (FPVs), while somatic mutations classified as germline by ISOWN are called false negative variants (FNVs).

One common theme across all tumor types tested is that FPVs are enriched with low VAF variants. For example, 23.8% of all FPVs in KIRC have VAF <20%, while just 0.52% of variants correctly predicted as germline have VAF <20%. In BRCA, 21% of all FPV versus 0.4% of all germlines have VAF <20%. For PAAD, the different is even more drastic: 55.4 versus 2.88%. This suggests that one source of classifications comes from unbalanced copy number variations affecting germline SNPs.

We detected 63.11% of all FPVs in PAAD in one sample only, whereas only 5.14% of true germline polymorphisms appear only once in the sample population. In KIRC, 87.81% of all FPVs are seen in a single sample, in contrast to 2.93% of germline polymorphisms. Similar ratios were observed in the other cancer types. These results indicate that the majority of the incorrectly predicted somatic mutations were called in single samples only. Because of this, these FPVs are unlikely to have a major effect on downstream analyses, as they would most likely be treated as low frequency passenger mutations.

Another interesting observation is that, in three out of six cancer sets, the gene most frequently involved with FPVs was MUC4. This gene accounted for 1.9% of all FPVs in BRCA, 3.5% in KIRC and 5.8% in COAD. This is significantly higher than expected by chance even after taking into account the gene length. According to Genecards (http://www.genecards.org/cgi-bin/carddisp.pl?gene=MUC4), this gene contains a region in the coding sequence which has a variable number (>100) of a 48-base tandem repeat. We hypothesize that the tandem repeat is responsible for mapping errors during the alignment and variant calling steps of upstream processing. The other genes affected by the same issue in at least one out of six datasets are MUC2, MUC6, and TTN, each of which contained tandem repeats and may be subject to similar issues. These observations highlight the fact that our classification method is not designed to identify sequencing errors and mapping artifacts. We recommend using ISOWN only after pre-filtering for possible artifacts (for example, sequencing and/or FFPE artifacts).

Turning to FNVs, one source of FNVs came from the classification of variants present in dbSNP/common_all but not in COSMIC as germline variants (Additional file 1: Table S3). Depending on the cancer type, between 0.9 and 9.3% of all FNVs are explained by this classification error. In addition, the VAFs for FNVs are significantly higher than the average VAF for all somatic mutations. For example, 38.8% of all FNVs in UCEC have VAF >40%, while only 20.7% of somatic mutations have VAF >40%. Because of this, FNV classification errors may be biased towards clonal driver somatic mutations that arise early in tumor development and have a high VAF, as well as oncogenes that are involved in amplification events. This is part of the rationale for the algorithm’s pre-processing step of labeling all known drivers with COSMIC CNT ≥100 as somatic and skipping the machine learning classification step.

One of the major concerns for proper somatic mutation classification is its accuracy with respect to the subset of “novel” variants that are catalogued by neither dbSNP/ExAC nor COSMIC. The ratio of novel variants among true somatic mutations ranges from 2.0% in COAD to 52.1% in PAAD. Interestingly, in five out of six cancer types, we find a smaller proportion of novel somatic mutations among the FNVs than among all somatic mutations, meaning that FNVs were depleted from novel mutations. For example, in the PAAD data set the percentage of novel variants dropped from 52.1% in all somatic mutations to 6% in FNVs (*p* value <0.0001 by Fisher proportional test). In the sixth cancer type (COAD), the FNV rate among novel and known somatic mutations was comparable. This means that ISOWN is no more likely to miss novel somatic mutations than it is to miss known ones.

### Application to cell lines

Cell lines represent a specific case for somatic mutation prediction where we expected a reduction in ISOWN performance. First, the number of samples are usually low (only two lines in the case presented below) and the sample frequency feature is not applicable. Second, because cell lines have cellularity close or equal to 100%, the VAF distribution for somatic and germline variants should show comparable patterns. In addition, the flanking region VAF feature may also be less relevant due to the high levels of cellularity. Thus, only seven out of ten features are fully applicable to this particular scenario.

VCF files with somatic and germline variants for the HCC1143 and HCC1954 breast cancer cell lines were downloaded from Cancer Genome Collaboratory (http://www.cancercollaboratory.org/). We used variants called using the DKFZ variant-calling pipeline (https://dockstore.org/containers/quay.io/pancancer/pcawg-dkfz-workflow) for the ICGC/TCGA PanCancer Analysis of Whole Genomes Project (https://dcc.icgc.org/pcawg). In this case, matching normal DNA (isolated from normal B lymphoblasts) was available to provide a gold standard for somatic mutations called from the cell lines. We considered only non-silent calls in coding regions, and the ratio of SNPs to somatic mutations was 8 to 1.

We trained NBC and LADTree using increasing numbers of TCGA BRCA (breast cancer) samples. Because of the limited number of cell lines, we removed the sample frequency feature from both the training and testing sets. The average recall across all training sets was 85% and the precision 63% (F1-measure 71.4%). We found that both NBC and LADTree had similar accuracies, but NBC generated more stable results with lower accuracy variance across the training sets (Additional file 2: Figure S8).

### Application to archival FFPE specimens

A major use case for ISOWN is the identification of somatic mutations in archival FFPE specimens, which often do not have accompanying blood or other normal tissue. To test the algorithm’s accuracy in this scenario, we sequenced 1491 estrogen receptor-positive (ER+) early breast cancer FFPE samples (see Additional file 1: Supplemental methods for more details) from the Tamoxifen versus Exemestane Adjuvant Mulitcentre (TEAM) clinical trial [41], which didn’t have matching normal tissues. ISOWN was used to call somatic SNVs in this set. To validate the call sets, the final list of TEAM somatic mutations was compared with three other publicly available breast cancer mutation sets (TGCA BRCA ER+ [42] and results published in [43]) to determine whether the somatic mutation frequency in each gene matched expectations.

Overall mutation loads in the genomic regions sequenced using our targeted sequencing panel were similar between TEAM samples and those from other data sources. We found no significant differences in gene mutation frequency between the ISOWN-processed TEAM samples and previously published breast cancer mutation frequencies using Fisher’s proportional test (false discovery rate >10%). For example, 30.5, 29.6, and 34.1% of samples contain mutations in the PIK3CA gene in the TEAM, TCGA BRCA, and Stephen et al. [43] sets, respectively. We also calculated the proportion of samples carrying at least one non-silent somatic mutation in each independent dataset. In the TEAM data set, 71.8% of samples carried at least one non-silent mutation, which is not significantly different from the 69.0% observed in the ER+ subset of breast cancer samples in TCGA BRCA, and 69.4% of ER+ samples in Stephen et al. (*p* value 0.558 from Fisher’s proportional test). In addition, the pattern of somatic mutations within genes matched the expected distribution.

Based on these three assessment criteria (mutational load, mutated gene frequency, and samples carrying at least one mutation) we conclude that the somatic mutation call set produced by ISOWN on a targeted FFPE sample set is comparable to the data sets produced by paired somatic mutation callers across three similar breast cancer data sets.

## Discussion

We describe the development and implementation of ISOWN, an accurate algorithm for discriminating germline polymorphisms from somatic mutations in cancer tissues in the absence of matching normal tissues. We achieved F1-measures ranging from 75.9–98.6% across multiple tumor types. The algorithm was validated using different sequencing strategies, including whole-exome sequencing and deep targeted sequencing, and different tissue types, including fresh frozen tumor tissues, cell lines, and FFPE samples.

The major challenge for this discrimination is the greatly unbalanced nature of the classification problem. After the various quality control and preprocessing steps, the number of germline polymorphisms is up to 500 times larger than somatic mutations, depending strongly on cancer type. ISOWN uses two mechanisms to overcome this imbalance. The first takes advantage of the fact that the vast majority of variants catalogued by dbSNP/common_all but not by COSMIC are germline polymorphisms. Removing this subset reduces the number of germline variants by roughly 70%, but the number of germline polymorphisms still greatly outweighs the somatic mutations. The second approach uses a data collapsing step in which we assume that any variant occurring in multiple samples is either somatic or germline. This assumption reduces the ratio of germline to somatic to 0.5–10 times depending on the cancer type.

The subsequent machine-learning classification step is based on ten different features, the most predictive of which are the three extrinsic features of the variants’ presence in the COSMIC, ExAC, and dbSNP databases, and the two intrinsic features sample frequency and VAF. As these databases grow and expand, we can expect the performance of the classifier to improve. In addition, because sample frequency is one of the strongest intrinsic features, the performance of the classifier improves as the number of samples in the training and testing sets increases. Interestingly, the predicted functional impact of the variant, while helpful in discriminating non-silent variants, is not essential for correct classification, as shown in the relatively good performance of the algorithm on silent mutations.

ISOWN was designed to accommodate multiple underlying supervised machine learning systems. Of the seven machine learning systems we evaluated, NBC and LADTree were consistently the best, achieving comparable accuracies across all cancer data sets. While there were no major differences between NBC and LADTree, the former is computationally faster.

We benchmarked ISOWN against six TCGA whole-exome sequencing datasets that had been generated using conventional matched normal sequencing and variant calling. The data sets varied both biologically (a range of mutational loads and mutational spectra) and technically (different paired variant callers and preprocessing steps). Using a set of ten features we were able to identify non-silent somatic mutations with an overall accuracy of ~99.5% across all six datasets. Cancer types with a high mutational load and a low germline:somatic ratio (COAD and UCEC) had the best performance, with an F1-measure ranging from 95–98%. Tumor types with a lower mutational load and a higher germline:somatic ratio (BRCA, ESO, and KIRC) had a reduced accuracy with F1-measures ranging from 85 to 93%. The worst performance was observed in PAAD (pancreatic adenocarcinoma), which has the highest germline:somatic ratio.

Some cancer driver prediction algorithms, for example, OncodriveCLUST [44], require a list of both non-silent and silent (synonymous) mutations. When applied to the task of predicting silent somatic mutations located in coding regions, ISOWN’s accuracy is reduced, but remains in the range of 69–97% (F1-measure). We have not evaluated ISOWN on whole genome sequences because several of the intrinsic features we use for discrimination, such as PolyPhen-2 functional impact, do not apply. In addition, COSMIC is currently heavily biased towards coding mutations obtained from exome sequencing studies, and the COSMIC CNT feature would bias the classifier away from non-coding somatic mutations.

In a recently published paper [45], nine somatic variant callers were evaluated and benchmarked against a set of high-confidence somatic mutations generated using alternative calling algorithms together with manual curation. Widely used paired somatic mutation callers such as Strelka [15] and MuTect [17] demonstrated the best sensitivity rates of ~83 and ~89%, respectively. When benchmarked against paired call sets, ISOWN demonstrates sensitivities ranging from 86.7% (for PAAD) to 98% for the rest of the datasets, indicating that ISOWN’s accuracy lies within the range that would be acceptable for the majority of research and clinical projects. The caveat, of course, is that ISOWN is trained against paired variant call sets from the appropriate tumor type, and its accuracy can never exceed that of the paired caller it is trained on. The variation in the number of germline SNPs per sample called by the different TCGA projects (Table 1) illustrates the strong effect that the choice of the paired variant calling pipeline may have on the training set.

The ISOWN algorithm works across multiple experimental designs, including whole-exome sequencing and targeted sequencing, and samples derived from fresh-frozen tissue, FFPE tissue blocks, and cell lines. For a large cohort of ER+ breast cancer patients with unpaired FFPE samples, ISOWN produced somatic mutation call rates that, on a per-sample and per-gene basis, were consistent with the values reported by several large paired sample studies of similar cohorts. In cell lines, we were able to predict somatic mutations in two breast cancer cell lines, achieving an F1-measure close to 75% when the classifier was trained on a breast cancer data set. The great majority of the cell lines registered with the Cancer Cell Line Encyclopedia (CCLE) portal are missing matching normal tissues, and only common germline polymorphisms are removed based on dbSNP and other external databases. Provided that an appropriate training set is used, ISOWN can be used for identifying somatic mutations in these cell lines.

ISOWN is applicable to two research scenarios. First is the case where a researcher has access to matched normal tissue for some, but not all, of the members of a cancer cohort. In this case, he or she will be able to call somatic mutations using a conventional paired variant caller like MuTect2. For the rest of the samples without matching normals, all variants including somatic and germlines are called in tumor-only mode using existing tools such as GATK or MuTect2*.* The somatic mutations are then used to train and validate ISOWN. Once trained and validated, ISOWN can be used to predict which of those variants called from the tumor-only samples are somatic mutations. Our benchmarks demonstrate that 25–50 samples are adequate for training ISOWN on highly mutated cancer types (>100 non-silent somatic mutations per sample), 50–100 samples for cancers with a moderate mutational load (10–100 non-silent somatic mutations per sample), and >100 samples for cancers with a high ratio of germline variants to somatic mutations (like PAAD). A researcher might also wish to reduce the overall cost of a cancer sequencing study by sequencing only sufficient matched normals to adequately train the classifier, and then using the classifier to call somatic mutations on unpaired tumor sequences obtained from the remainder of the donors.

The second research scenario is where no matched normal tissue is available at all, either because it was never collected (e.g., cell lines, pathology archives) or because donor consent was obtained in a narrow fashion that forbids examination of the germline. In such cases, ISOWN can be trained on a reference data set that has similar biology to the cohort of interest. For example, we demonstrate that ISOWN’s accuracy is degraded but still usable when the classifier is trained on one tumor type and then tested with another that has a similar mutational load (F1-measure 98% for training with COAD and testing with UCEC). Even in the worst case, in which paired variant calls from breast cancer primaries were used to train the classifier to detect somatic mutations in two breast cancer cell lines, still had an accuracy in the 70% range (F1 measure). For convenience, we have included six standard training sets in the ISOWN software package.

Like many other software, ISOWN also has a few limitations. First, its accuracy suffers with cancers with low mutational load and small sample sets. Second, the algorithm isn’t trained to recognize sequencing artifacts related to FFPE damage or other artifacts; these must be removed via upstream filters prior to the classification task. Third, for best results the algorithm requires a set of 25–100 samples to train the classifier; one of the standard training sets provided with ISOWN can be used, but accuracy might be moderately reduced. Fourth, the algorithm has only been tested on variants that fall in coding regions and is unlikely to work on whole genomes until the databases of somatic mutations become more comprehensive. Lastly, the current version of ISOWN is not set up to call small insertions/deletions (indels), a task that is challenging due to the high rate of sequencing and mapping artifacts that contribute to indel calls, and their relative scarcity. These challenges will be addressed in the next releases of ISOWN.

Future work will focus on improving the classifier performance for cancer types with low mutation frequencies, datasets with low numbers of samples, indels, and non-coding mutations. In addition, we plan to add additional reference training sets to the ISOWN package.

## Conclusions

In this work we have presented a novel and accurate computational algorithm called ISOWN for predicting somatic mutations from cancer tissues in the absence of matching normal samples. ISOWN uses machine learning and external databases along with the sequencing characteristics information retrieved from the samples themselves. ISOWN was extensively validated across six different cancer types with different mutation loads where F1-measures range from 75.9 to 98.6%. In addition, ISOWN was tested on FFPE, fresh frozen, and cell line tissues.

ISOWN can help researchers to accelerate sequencing process, reduce financial investment in sample sequencing and storage requirements, or increase the power of analysis by increasing the number of tumor samples sequenced with the same resources. In addition, ISOWN is useful in cases where the patient consent prevents normal tissue collection or when a study is based on retrospective biopsies where normal tissues were not collected. ISOWN is freely available on GitHub together with a detailed manual of how to install and use it.

## Availability and requirements

Project name: ISOWN (Identification of Somatic mutations Without Normal tissues)

Project home page: https://github.com/ikalatskaya/ISOWN

Operating system(s): Linux, iOS

Programming language: C, Perl, Java

Other requirements: Tabix, Annovar, Weka

License: GNU

Any restrictions to use by non-academics: please contact the authors

## Additional files

Additional file 1: Supplemental material including supplementary methods and supplementary results as well as **Tables S1** and **S3–** **S8**. **Table S1:** Results from 10-fold cross-validation. **Table S3:** Comparison of the somatic mutation ratio in the whole dataset vs in the subset of variants that were catalogued by dbSNP/common_all but not by COSMIC. **Table S4:** Number of germline variants with high CNT in different cancer sets. **Table S5:** Number of variants with “mixed” labels in different cancer sets. **Table S6:** Uneven distribution of the somatic mutations and germline polymorphisms in Mutation Assessor categories. **Table S7:** Uneven distribution of the somatic mutations and germline polymorphisms in PolyPhen-2 categories. (PDF 208 kb)

Additional file 2: Figures S1–S8. **Figure S1:** Concent of flanking regions.** Figure S2:** Mono-labeled approach expanation: variant labels are allele- specific, not sample specific. **Figure S3:** VAF density distribution for different cancer sets. **Figure S4:** Sample frequencies for somatic mutations and germline polymorphisms. **Figure S5:** ISOWN validation (False Positive Rate). **Figure S6:** ISOWN testing on silent variants (F1-measure). **Figure S7:** ISOWN validation on variants with different VAF (F1-measure). **Figure S8:** ISOWN validation on cell lines. (PDF 18546 kb)

Additional file 3: Table S2. Table giving the ISOWN performance measures (F1-measure, Recall, False Positive Rate, Precision and Accuracy) calculated based on held-out independent sample set across six cancer datasets. (XLSX 125 kb)

## Abbreviations

BRCA: Breast invasive carcinoma
CARNAC: Consensus And Repeatable Novel Alterations in Cancer
CI: Confidence interval
COAD: Colon adenocarcinoma
ER: Estrogen receptor
ESO: Esophageal adenocarcinoma
ExAC: Exome Aggregation Consortium
FFPE: Formalin-fixed paraffin embedded
FNV: False negative variant
FPR: False positive rate
FPV: False positive variant
KIRC: Kidney renal clear carcinoma
NBC: naïve Bayes classifier
PAAD: Pancreatic adenocarcinoma
SNP: Single nucleotide polymorphism
SNV: Single nucleotide variant
SVM: Support vector machine
TCGA: The Cancer Genome Atlas
UCEC: Uterine corpus endometrial carcinoma
VAF: Variant allele frequency
