# Supplementary material for: ISOWN: accurate somatic mutation identification in the absence of normal tissue controls
Source: Genome Med. 2017 Jun 29;9:59. doi: 10.1186/s13073-017-0446-9 (PMC5490163; doi:10.1186/s13073-017-0446-9)
Supplement: Supplementary file 1 — Supplemental material including supplementary methods and supplementary results as well as Tables S1 and S3– S8. Table S1: Results from 10-fold cross-validation. Table S3: Comparison of the somatic mutation ratio in the whole dataset vs in the subset of variants that were catalogued by dbSNP/common_all but not by COSMIC. Table S4: Number of germline variants with high CNT in different cancer sets. Table S5: Number of variants with “mixed” labels in different cancer sets. Table S6: Uneven distribution of the somatic mutations and germline polymorphisms in Mutation Assessor categories. Table S7: Uneven distribution of the somatic mutations and germline polymorphisms in PolyPhen-2 categories. (PDF 208 kb) [file 13073_2017_446_MOESM1_ESM.pdf]

Additional File 1 - Supplemental material

ISOWN: accurate somatic mutation identification in the absence of normal tissue controls

SUPPLEMENTAL METHODS:

List of tested learning algorithms: We have tested the performance of different learning algorithms that represent major classification methods:

(i) The rule-based method generate classification models using a collection of "if ... then ..." rules. It is also known as *Separate-And-Conquer* method. This method is generating a rule that covers a subset of the training examples and then removing all examples covered by the rule from the training set. This process is repeated iteratively until there are no examples left to cover. The algorithms are usually computationally inexpensive, are capable of incorporating categorical and continuous variables and the developed models are usually easy to interpret. RIPPER, a deterministic rule-based classifier algorithm, was evaluated in the current study. RIPPER states for "Repeated Incremental Pruning to Produce Error Reduction" and is named JRIP (i.e. Java implementation of RIPPER) in WEKA. JRip builds a ruleset by repeatedly adding rules to an empty ruleset until all positive examples are covered (1). After the building process, a ruleset is optimized to reduce its size and improve its fit to the training data. It helps to prevent overfitting.

(ii) *Decision tree algorithm* is a very popular and practical approach for pattern classification. Decision tree is constructed generally in a greedy, top down recursive manner. Three algorithms that belong to this approach were tested:

a. J48 is the Weka implementation of C4.5 algorithm, the most popular tree classifier (2). At each node of the tree, C4.5 chooses the attribute of the data that most effectively splits attribute set into subsets enriched in one class or the other. The splitting criterion is the normalized information gain. The attribute with the highest normalized information gain is chosen to make the decision. The C4.5 algorithm then recurs on the smaller sub-lists. No changes to the default parameters were made.

b. Random Forest (3) is a well-known meta-learner that generates many individual trees. Each tree depends on the values of a random vector independently sampled and with the same distribution for all trees in the forest. For forests, the generalization error converges to a limit as the number of trees in the forest becomes larger. The main advantages are related to its robustness to noise, and fast computation

over large datasets. Number of trees was set to 100 for all datasets and number of features was set to 3 (calculated as a square root of the whole number of attributes).

c. A Least Absolute Deviation (LADTree) is one of the decision tree machine learning algorithm. The LADTree algorithm applies logistic boosting algorithm in order to induce an alternating decision tree. It uses least absolute deviation (LAD) to find the error criterion to obtain regression trees (4). Default parameters were used in all experiments.

(iii) We tested two *function-based algorithms*: Support Vector Machine (called SMO in WEKA and denoted SVM in this study) and Logistic Regression. SVM learner used a linear kernel that showed a better performance in comparison to RBF kernel. Complexity parameter (C) for SVM and ringe (R) parameter for Logistic regression were optimized for each cancer set separately using WEKA meta-classifier CVParameterSelection. SVM is a classifier that converts data objects into a multi-dimensional vector and defines a separating hyperplane among the objects belonging to different classes.

(iv) *Naïve Bayes Classifier (NBC)* is known as a simple probabilistic classifier and assumes the independence of features given a class. NBC was tested with and without Kernel Density Estimation (KDE) and with and without supervised discretization (SD) to process numeric attributes. KDE might improve the performance if the normality assumption of numeric value distribution is grossly incorrect. Handling numeric attributes using SD might also influence the final output from the classifier. Validation showed that NBC with SD set to ‘true’ and KDE set to ‘false’ shows the most accurate results.

(v) In *distance-based methods* (also called instance-based methods *or* lazy learning) a distance function is used to determine which member of the training set is closest to an unknown test instance. We tested IB1 (Basic nearest-neighbor instance-based learner) and IBk (k-nearest-neighbors classifier), but due to slowness and very poor performance these classifiers were excluded from further validation.

FFPE sample sequencing and processing: 1491 ER+ early breast cancer FFPE samples from the Tamoxifen versus exemestane adjuvant mulitcentre (TEAM) clinical trial were sequenced using a breast cancer specific gene panel sized ~0.55 Mbps using AmpliSeq technology. Raw reads were aligned

against hg19 using novoalign (version 2.07.14) and only reads that aligned uniquely (mapping qualities > 30) were kept for downstream analysis. Aligned reads were then subjected to local realignment and base quality recalibration prior to calling variant calls with GATK's UnifiedGenotyper (version 1.3.16) with downsampling disabled. Low confidence variants were removed with the following filters: (a) read depth >= 50; (b) maximum number of variants per 10 bp window = 3; (c) strand bias > -10; and (d) variant quality >= 50.

SUPPLEMENTAL RESULTS:

Justification of the variant pre-labeling stage: The variants that are catalogued by dbSNP/common_all but not by COSMIC are significantly depleted from somatic mutations. The percent of somatic variants in this subset is consistent ranging from 0.01 to 0.02% (Additional file 1: suppl. Table 3). That's a reason we named this subset as a "gold standard negative set". Rather than going through the classifier, each variant of this type was *a priori* labeled as a germline. For example, in COAD only roughly one mutation per sample (245 somatic mutations across 215 samples) that are catalogued by dbSNP/common_all but not by COSMIC will classified as germline polymorphisms. These misclassified somatic variants are slightly increasing the final false negative rate, but this assumption significantly improves the overall performance of the classifier, first because 1,374,557 variants are classified correctly; secondly, because removing this huge pile of the germlines from the testing set balances positive and negative instances and again improving the final performance of the classifier.

A majority of the variants catalogued by COSMIC were identified only in one sample (CNT=1). But in a very few special cases, CNT might go up to several hundreds or higher (like for the variant chr3,178952085A>G in PIK3CA gene CNT=1,635 or for chr7,140453136A>T variant in BRAF CNT=19,966). They are well known cancer-associated and, in many cases, also cancer-causing variants. Vast majority of those in the investigated datasets are somatic and only in the very rare cases could be germline: out of ~9,000,000 germline polymorphisms from ~1,000 samples analyzed in this study only 7 have CNT >=100 (Additional file 1: suppl. Table 4). In counterweight to "gold standard negative set" these variants were called "gold standard positive set". All variants with CNT>=100 were labeled as somatic and bypassed the classifier (Figure 1). This filtering step helped us to accomplish a number of tasks: (1) we are sure that the classifier is not missing the most valuable for further analysis variants; (2) classifier is not confused by extreme outliers in CNT feature; (3) final true positive rate (recall) might be improved.

Variants are monolabeled across all tumor samples: We made an assumption that variants that are sharing the same genomic position and allelic set is either somatic or germline across all tumour samples within a particular cancer data set. To justify this assumption we calculated the number of unique variants

that have been labeled as both somatic and germline in different samples using conventional paired sequencing, or, as we call them, “mixed variants”. Additional file 1: Suppl. Table 5 shows that all datasets have a comparable rate of mixed labeled variants ranging 0.005-0.70% of all unique variants in the set. Interesting that PAAD set contain only five variants with mixed labels.

Number of mixed labels doesn’t correlate either with number of samples in the dataset nor with a ratio of somatic nor with somatic mutational load and might represent an internal property of the set.

A significant portion of all mixed labeled variants are called only in two samples (in one – somatic, and in another – germline) [Additional file 1: suppl. table 5]. As the sample frequency rate for these mutations is low, the effect of the simplifying assumption won’t have large impact of the final classification output. Another telling observation is that a significant portion of all variants with mixed labels were called by the TCGA projects in at least 50 samples and in all but one sample this variant was labeled as germline. This might indicate a misclassification error by the paired mutation caller (Additional file 1: suppl. Table 5).

‘Mixed label’ variants were excluded from training and testing sets.

REFERENCES

1. Cohen WW. Fast effective rule induction. *Proceedings of the twelfth international conference on machine learning* 1995; 115-123.

2. Quinlan RC. 4.5: Programs for machine learning Morgan Kaufmann Publishers Inc. *San Francisco, USA* 1993.

3. Breiman L. Random Forests. Statistics Department, University of California. *Machine learning* 2001.

4. Holmes G, B. Pfahringer, R. Kirkby, E. Frank, and M. Hall. Multiclass alternating decision trees. *European Conference on Machine Learning* 2002; 161-172.

SUPPLEMENTARY TABLE

Suppl. Table 1. Performance measures from 10-fold cross-validation using seven classification algorithms performed on randomly generated 1000 training sets each containing 700 somatic mutations and 700 germline polymorphisms from six different cancer types.

Suppl. Table 2: Performance measures calculated based on held-out independent sample set across six cancer datasets. NBC and LADTree algorithms were chosen in the 10-fold cross-validation and used here. Classifiers were trained based on the gradually increasing number of samples (Samples In Training Set). Number of positive instances collected from the indicated number of randomly selected samples is shown in Column B. Provided as an excel file (additional file 3).

Suppl. Table 3. Comparison of the somatic mutation ratio in the whole dataset vs in the subset of variants that were catalogued by dbSNP/common_all but not by COSMIC. The latest contains vanishingly small number of somatic mutations.

Suppl. Table 4. Number of germline variants with high CNT in different cancer sets.

Suppl. Table 5. Number of variants with “mixed” labels in different cancer sets as well as their characteristics. *Only non-silent SNVs in coding regions.

Suppl. Table 6. Distribution of the collapsed (unique) somatic mutations and germline polymorphisms in different categories for functional impacts based on Mutation Assessor (MA) annotations across six cancer datasets. Only variants with known MA annotations were taken into account. Germlines are prone to be more neutral, whereas somatic mutations have more high and medium impacts on the protein functionality. Mutation Assessor serves as an independent feature in ISOWN. The p-value was estimated based on 2-sample test for equality of proportions.

Suppl. Table 7. Distribution of the collapsed (unique) somatic mutations and germline polymorphisms in three categories of PolyPhen-2 across six cancer datasets. Only variants with known annotations were taken into account. Germlines are significantly enriched in ‘benign’ type, and somatic in both ‘probably’ and ‘possibly damaging’. PolyPhen-2 also serves as an independent feature in ISOWN.

SUPPL. TABLE 1

Results from 10-fold cross-validation using seven classification algorithms that was performed on randomly generated 1000 training sets each containing 700 somatic mutations and 700 germline polymorphisms from six different cancer types.

Cancer	Classifier	F1-measure	Recall	FPR	Precision	AUC
UCEC	JRip	97.94%	98.05%	2.17%	97.84%	98.69%
COAD	JRip	96.34%	96.71%	4.06%	95.98%	97.58%
KIRC	JRip	97.47%	97.40%	2.45%	97.55%	98.23%
BRCA	JRip	96.69%	97.24%	3.90%	96.15%	97.00%
ESO	JRip	94.04%	95.60%	7.73%	92.54%	95.29%
PAAD	JRip	96.04%	96.51%	4.47%	95.58%	97.05%
UCEC	Random Forest	98.20%	98.16%	1.77%	98.23%	99.84%
COAD	Random Forest	96.46%	96.94%	4.06%	95.99%	99.32%
KIRC	Random Forest	97.50%	97.26%	2.25%	97.74%	99.66%
BRCA	Random Forest	96.45%	96.22%	3.30%	96.69%	99.06%
ESO	Random Forest	93.07%	94.08%	8.08%	92.09%	97.25%
PAAD	Random Forest	96.16%	96.79%	4.52%	95.54%	99.11%
UCEC	J48	97.80%	98.06%	2.47%	97.55%	98.47%
COAD	J48	96.16%	96.55%	4.25%	95.79%	97.49%
KIRC	J48	97.42%	97.25%	2.39%	97.60%	98.25%
BRCA	J48	96.57%	97.34%	4.25%	95.83%	96.77%
ESO	J48	93.77%	95.29%	7.96%	92.32%	95.56%
PAAD	J48	95.90%	96.57%	4.83%	95.24%	96.28%
UCEC	Logistic Regression	97.48%	97.40%	2.45%	97.55%	99.42%
COAD	Logistic Regression	95.17%	95.27%	4.93%	95.08%	98.65%
KIRC	Logistic Regression	95.94%	95.63%	3.72%	96.25%	99.02%
BRCA	Logistic Regression	95.62%	95.25%	3.99%	95.98%	98.47%
ESO	Logistic Regression	92.11%	93.05%	9.00%	91.19%	95.85%
PAAD	Logistic Regression	95.36%	95.84%	5.16%	94.89%	98.46%
UCEC	LADTree	98.29%	98.23%	1.64%	98.36%	99.84%
COAD	LADTree	96.59%	96.84%	3.68%	96.34%	99.40%
KIRC	LADTree	97.71%	97.61%	2.19%	97.81%	99.69%
BRCA	LADTree	96.81%	97.03%	3.42%	96.60%	99.10%
ESO	LADTree	94.53%	95.45%	6.51%	93.62%	97.88%
PAAD	LADTree	96.38%	96.92%	4.19%	95.86%	99.05%
UCEC	Naive Bayes	98.29%	97.93%	1.34%	98.65%	99.81%
COAD	Naive Bayes	96.11%	95.06%	2.75%	97.19%	99.39%
KIRC	Naive Bayes	96.58%	95.02%	1.74%	98.20%	99.56%

BRCA	Naive Bayes	96.40%	95.84%	3.00%	96.96%	99.01%
ESO	Naive Bayes	89.47%	90.45%	11.75%	88.51%	95.69%
PAAD	Naive Bayes	95.13%	94.32%	3.97%	95.97%	98.93%
UCEC	SVM	97.41%	97.26%	2.43%	97.57%	97.42%
COAD	SVM	94.06%	94.24%	6.12%	93.90%	94.06%
KIRC	SVM	95.51%	94.96%	3.88%	96.07%	95.54%
BRCA	SVM	94.69%	92.81%	3.23%	96.64%	94.79%
ESO	SVM	89.33%	90.17%	11.71%	88.51%	89.23%
PAAD	SVM	95.68%	96.41%	5.12%	94.96%	95.64%

SUPPL. TABLE 2

Available in .xlsx format (Additional file 3)

SUPPL. TABLE 3

Comparison of the somatic mutation ratio in the whole dataset vs in the subset of variants that were catalogued by dbSNP/common_all but not by COSMIC. The latest contains vanishingly small number of somatic mutations. In contrast, significant portion of somatic mutations catalogued.

Dataset [Source]	Total number of variants in the set, germline / somatic [% of somatic]	Number of variants catalogued by dbSNP/common but not COSMIC, germline/somatic [% of somatic]
UCEC [TCGA]	504,241 / 38,012 [7.0%]	368,834 / 80 [0.02%]
COAD [TCGA]	1,932,510 / 60,624 [3.04%]	1,374,557 / 245 [0.017%]
KIRC [TCGA]	2,416,155 / 10,489 [0.43%]	1,744,218 / 371 [0.02%]
ESO [dbGAP]	790,051 / 26,098 [3.19%]	550,897 / 66 [0.012%]
PAAD [TCGA]	1,263,918 / 5,593 [0.44%]	879,313 / 87 [0.01%]
BRCA [TCGA]	1,037,432 / 5,556 [0.53%]	751,453 / 77 [0.01%]

SUPPL. TABLE 4

Number of germline variants with high CNT in different cancer sets.

Dataset	Number of germlines with CNT >= 150	Number of germlines with CNT >= 100	Number of germlines with CNT >= 50	Number of germlines with CNT >= 30
UCEC [TCGA]	0	0	0	41
COAD [TCGA]	1	1	1	61
KIRC [TCGA]	1	1	1	146
ESO [dbGAP]	2	2	3	46
PAAD [TCGA]	1	1	1	73
BRCA [TCGA]	3	3	4	78

SUPPL. TABLE 5

Number of variants with “mixed” labels in different cancer sets as well as their characteristics. *Only non-silent SNVs in coding regions.

Dataset	Total number of the unique variants in the dataset*	Total number of the unique variants with mixed labels [% of total]	Number of mixed variants with `1:50+` pattern	Number of mixed variants with “1:1” pattern
UCEC [TCGA]	74,258	126 [0.17%]	34	35
COAD [TCGA]	160,818	1,127 [0.70%]	263	363
KIRC [TCGA]	118,119	662 [0.56%]	355	67
ESO [dbGAP]	81,369	339 [0.42%]	52	93
PAAD [TCGA]	79,437	5 [0.006%]	2	0
BRCA [TCGA]	58,061	195 [0.330%]	83	15

SUPPL. TABLE 6

Distribution of the collapsed (unique) somatic mutations and germline polymorphisms in different categories for functional impacts based on Mutation Assessor (MA) annotations across six cancer datasets. Only variants with known MA annotations were taken into account. Germlines are prone to be more neutral, whereas somatic mutations have more high and medium impacts on the protein functionality. Mutation Assessor serves as an independent feature in ISOWN. The p-value was estimated based on 2-sample test for equality of proportions.

Cancer	Annotation from Mutation Assessor	Germline, %	Somatic, %	p-value from prop test
KIRC	High	2.599 %	6.036 %	<1.0E-15
KIRC	Medium	23.299 %	32.134 %	<1.0E-15
KIRC	Low	34.182 %	34.198 %	>0.05
KIRC	Neutral	39.921 %	27.632 %	<1.0E-15
COAD	High	2.444 %	5.946 %	<1.0E-15
COAD	Medium	22.394 %	34.239 %	<1.0E-15
COAD	Low	33.462 %	34.222 %	0.018
COAD	Neutral	41.700 %	25.592 %	<1.0E-15
UCEC	High	2.188 %	5.466 %	<1.0E-15
UCEC	Medium	19.832 %	34.335 %	<1.0E-15
UCEC	Low	32.633 %	35.833 %	<1.0E-15
UCEC	Neutral	45.347 %	24.366 %	<1.0E-15
ESO	High	2.934 %	7.145 %	<1.0E-15
ESO	Medium	23.343 %	35.239 %	<1.0E-15
ESO	Low	33.155 %	33.740 %	>0.05
ESO	Neutral	40.568 %	23.876 %	<1.0E-15
BRCA	High	2.594 %	6.303 %	<1.0E-15
BRCA	Medium	21.404 %	33.944 %	<1.0E-15
BRCA	Low	33.686 %	34.710 %	0.640
BRCA	Neutral	42.316 %	25.043 %	<1.0E-15

PAAD	High	2.491 %	5.293 %	<1.0E-15
PAAD	Medium	22.073 %	34.478 %	<1.0E-15
PAAD	Low	33.163 %	34.371 %	0.021
PAAD	Neutral	42.272 %	25.858 %	<1.0E-15

SUPPL. TABLE 7

Distribution of the collapsed (unique) somatic mutations and germline polymorphisms in three categories of PolyPhen-2 across six cancer datasets. Only variants with known annotations were taken into account. Germlines are significantly enriched in 'benign' type, and somatic in both 'probably' and 'possibly damaging'. PolyPhen-2 also serves as an independent feature in ISOWN.

Cancer	Annotation from PolyPhen-2	Germline, %	Somatic, %	p-value from prop.test
KIRC	Benign	55.672 %	38.797 %	<1.0E-15
KIRC	Probably damaging	28.663 %	43.418 %	<1.0E-15
KIRC	Possibly damaging	15.649 %	17.764 %	0.001
COAD	Benign	57.097 %	34.870 %	<1.0E-15
COAD	Probably damaging	27.133 %	48.526 %	<1.0E-15
COAD	Possibly damaging	15.743 %	16.589 %	0.0025
UCEC	Benign	63.540 %	37.531 %	<1.0E-15
UCEC	Probably damaging	21.614 %	43.041 %	<1.0E-15
UCEC	Possibly damaging	14.824 %	19.428 %	<1.0E-15
ESO	Benign	55.844 %	32.519 %	<1.0E-15
ESO	Probably damaging	28.601 %	50.315 %	<1.0E-15
ESO	Possibly damaging	15.544 %	15.544 %	0.000078
BRCA	Benign	60.111 %	38.406 %	<1.0E-15
BRCA	Probably damaging	23.934 %	43.665 %	<1.0E-15
BRCA	Possibly damaging	15.941 %	17.928 %	0.01
PAAD	Benign	58.301 %	36.792 %	<1.0E-15
PAAD	Probably damaging	26.414 %	46.780 %	<1.0E-15
PAAD	Possibly damaging	15.259 %	16.415 %	0.01
