# Supplementary material for: ISOWN: accurate somatic mutation identification in the absence of normal tissue controls
Source: Genome Med. 2017 Jun 29;9:59. doi: 10.1186/s13073-017-0446-9 (PMC5490163; doi:10.1186/s13073-017-0446-9)
Supplement: Supplementary file 2 — Figure S1: Concent of flanking regions. Figure S2: Mono-labeled approach expanation: variant labels are allele- specific, not sample specific. Figure S3: VAF density distribution for different cancer sets. Figure S4: Sample frequencies for somatic mutations and germline polymorphisms. Figure S5: ISOWN validation (False Positive Rate). Figure S6: ISOWN testing on silent variants (F1-measure). Figure S7: ISOWN validation on variants with different VAF (F1-measure). Figure S8: ISOWN validation on cell lines. (PDF 18546 kb) [file 13073_2017_446_MOESM2_ESM.pdf]

Additional File 2 - Supplemental Figures

ISOWN: accurate somatic mutation identification in the absence of normal tissue controls

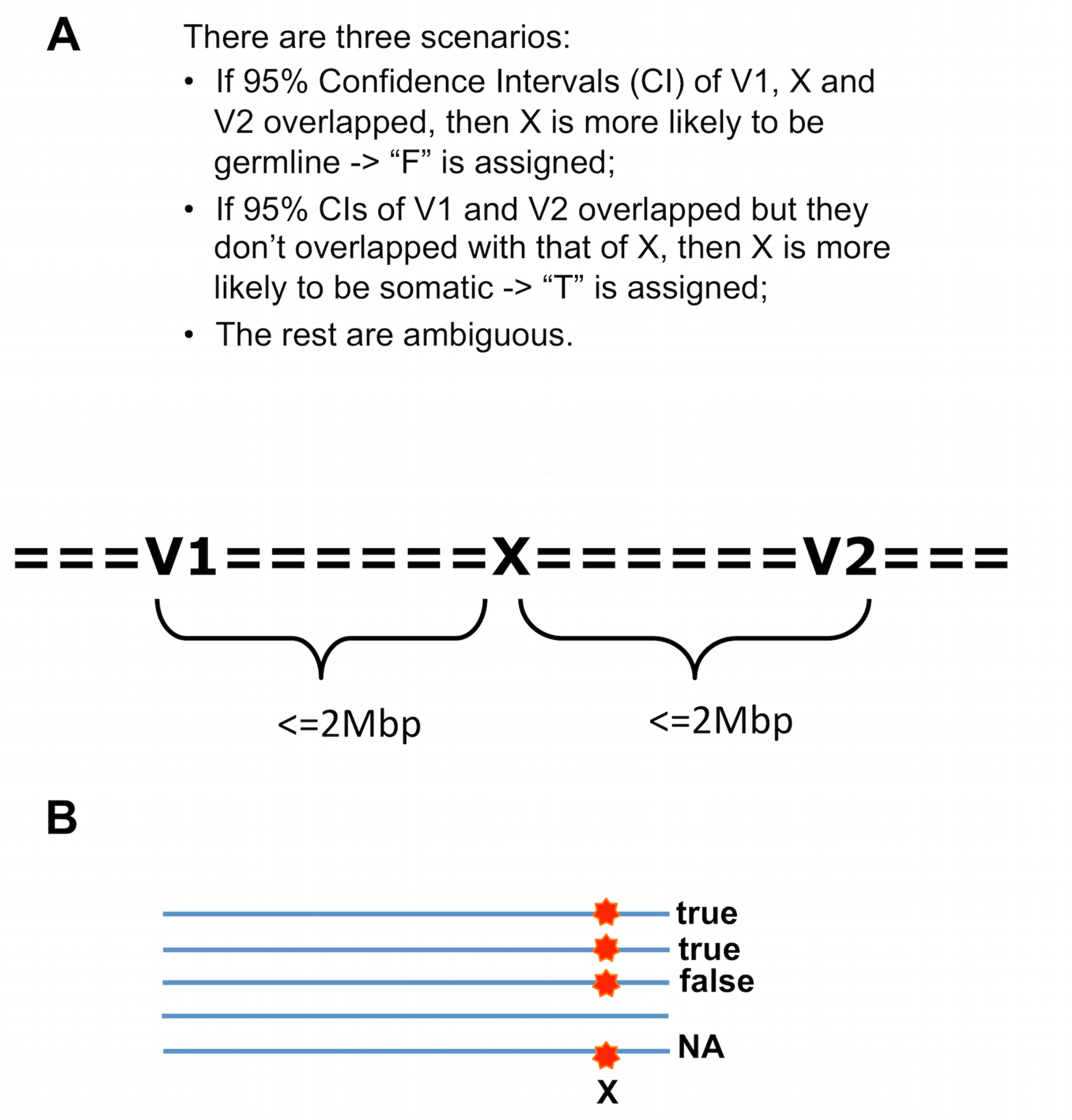

Suppl. Figure 1. Flanking Regions. (A) Concept of flanking region calculation; (B) Example of the usage of flanking region estimation in the classifier. Let’s assume four samples out of five contain a mutation X. Flanking region was estimated for all four cases and determined as “false”, “true”, “true” and “NA”. 50% of samples are positive. Flanking region for this variant in the collapsed set is equal 0.5.

Additional File 2 - Supplemental Figures

ISOWN: accurate somatic mutation identification in the absence of normal tissue control

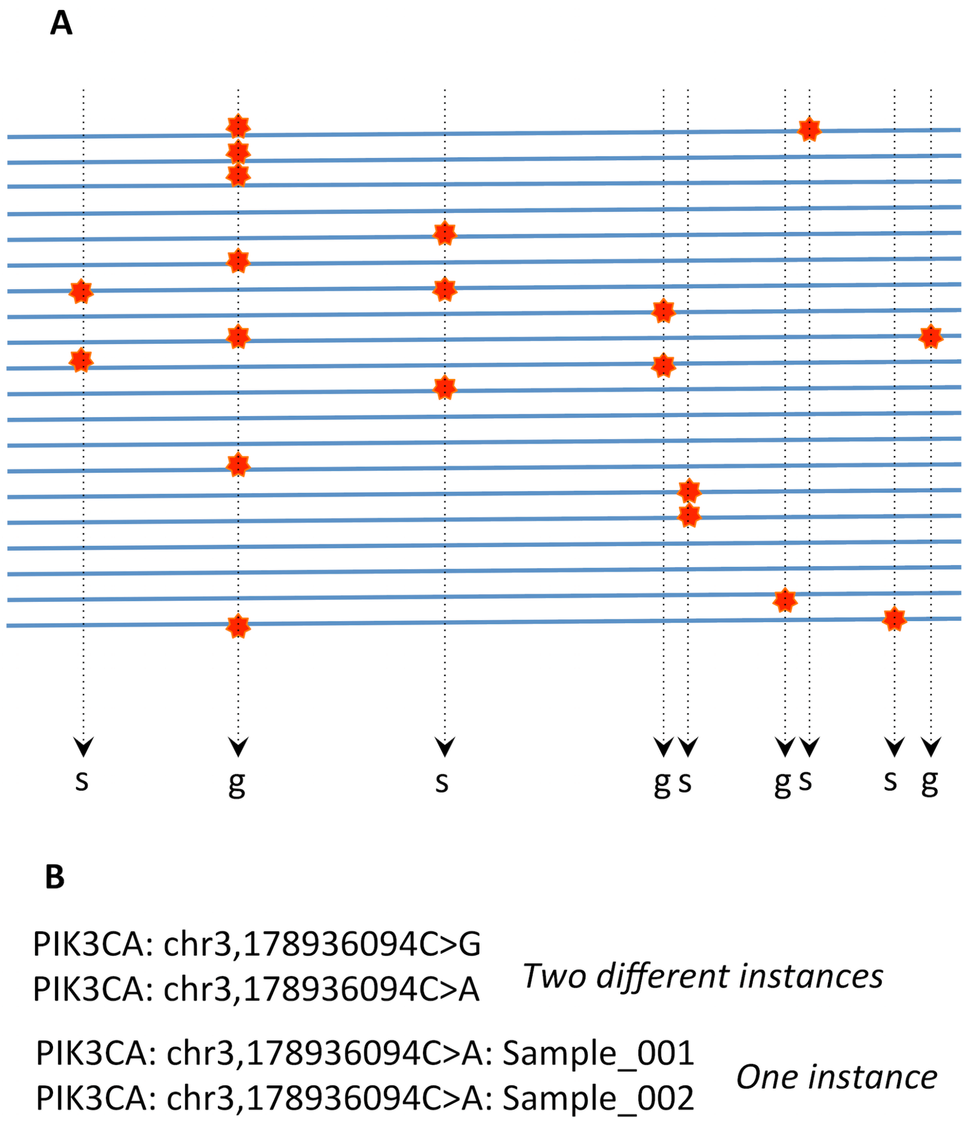

Suppl. Figure 2. Labels are allele-specific, not sample specific. (A) Let's assume that we have a number of sequenced genomes (represented by blue lines) with a number of called variants (represented by red stars). Number of "unique variants" in this case is equal 9 – total number of positions, total number of variants is equal a total number of red stars on the picture (20). We made an assumption that a variant (the same genomic position and the same alternative allele) called across several tumor samples are all either germlines (g) or somatic (s). (B) Examples: two mutations in PIK3CA at the same genomic position but resulting in different alternative alleles accounts as two different instances, whereas two identical mutations in two different samples accounts as one instance.

Additional File 2 - Supplemental Figures

ISOWN: accurate somatic mutation identification in the absence of normal tissue controls

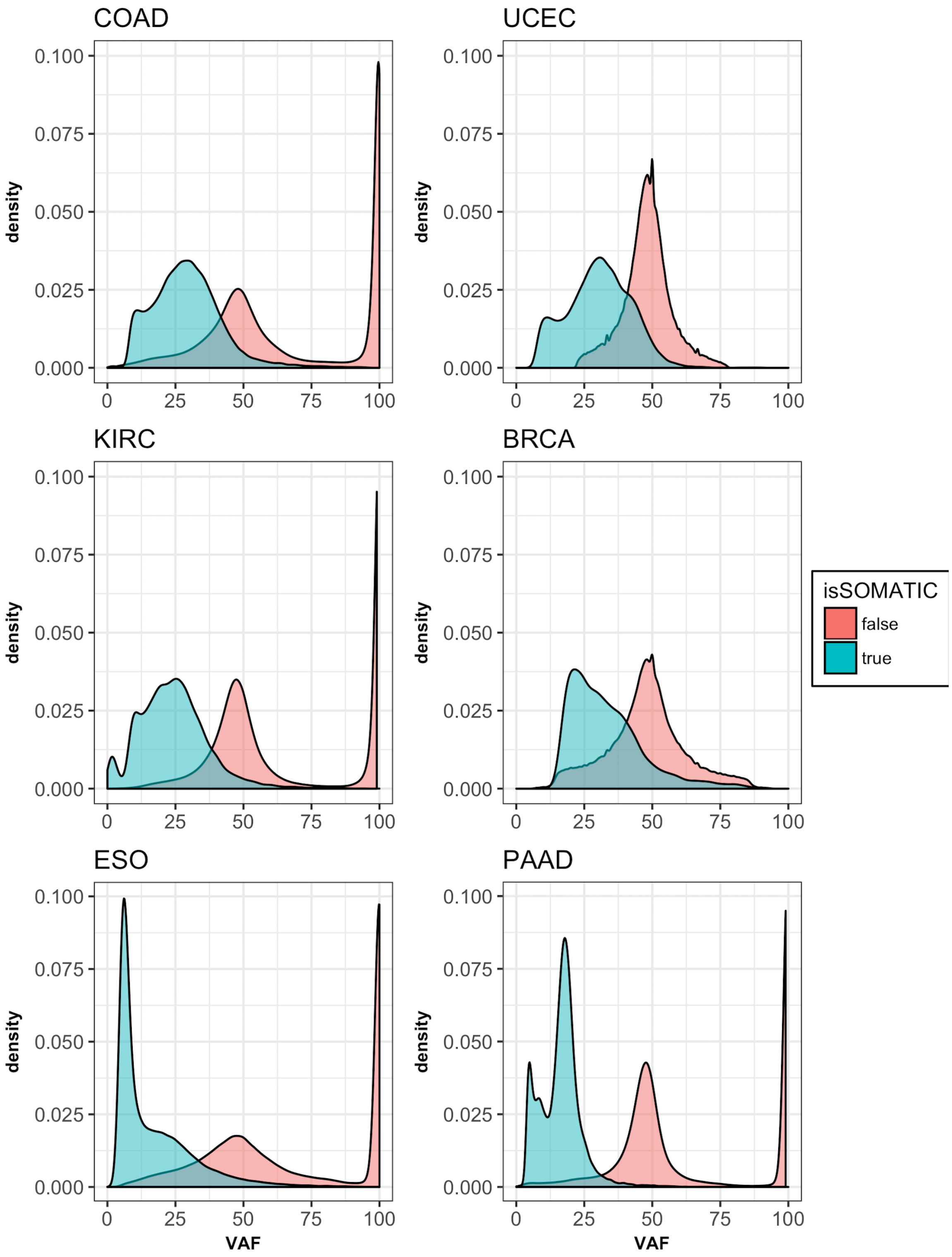

Suppl. Figure 3. VAF density distribution for different cancer sets.

Additional File 2 - Supplemental Figures

ISOWN: accurate somatic mutation identification in the absence of normal tissue control

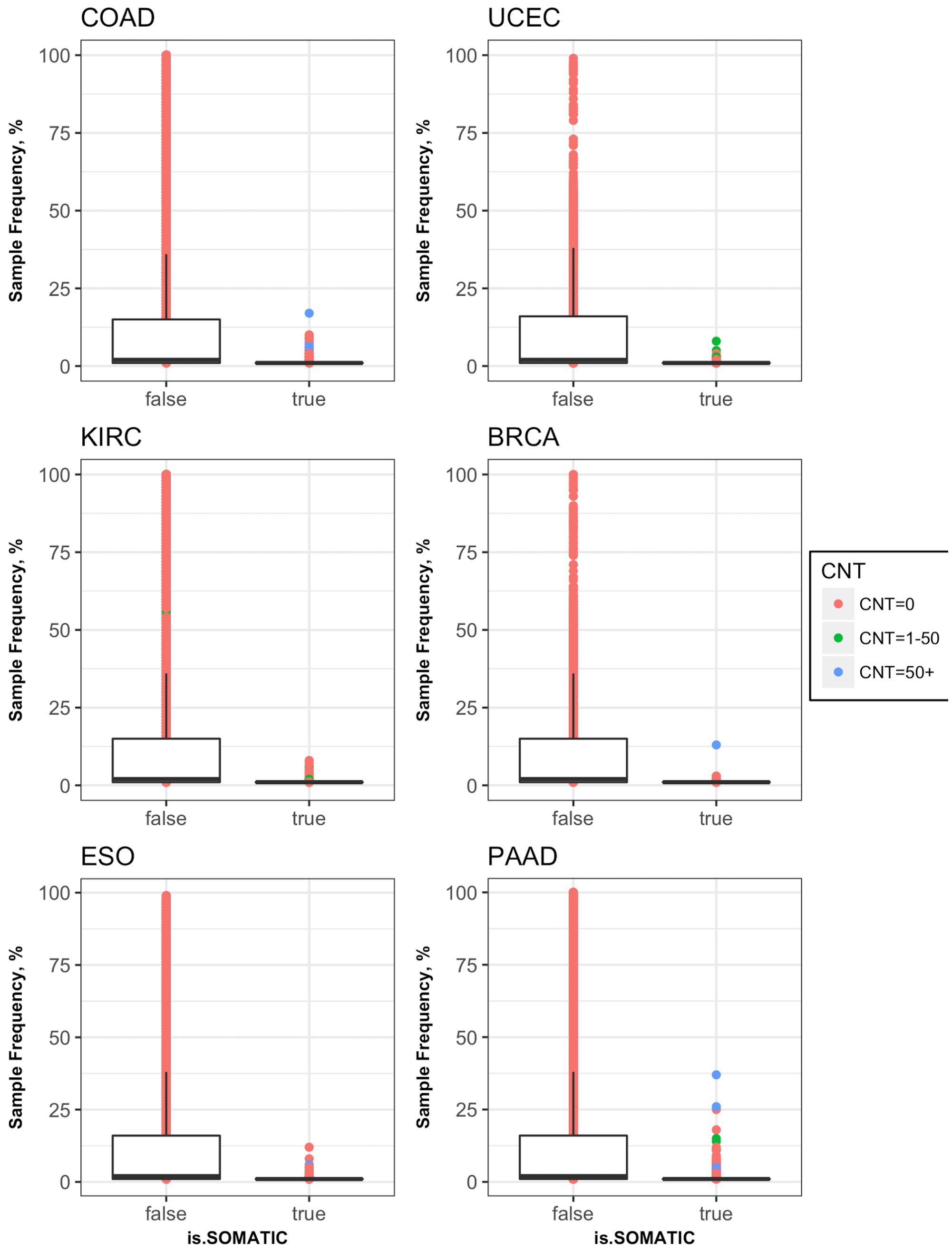

Suppl. Figure 4. Sample frequencies for somatic mutations and germline polymorphisms calculated based on 100 randomly selected samples from six whole-exome sequencing cancer datasets. The shapes of the boxplots show that 75% of all germline variants in all six cancer sets have sample frequency less than 15-16%. At the same time, the maximal sample frequency for germlines reaches 100% across all cancer sets, whereas maximal somatic mutation sample frequency is equal 40% in PAAD (KRAS mutation), 15% in BRCA (mutation in PIK3CA), 11% in ESO and COAD, 7% in KIRC and UCEC. Thus, sample frequency calculated for each variant might help to distinguish somatic mutations and germline polymorphisms with high frequency. The figure also indicates that in four out six cancer sets somatic mutations with the highest sample frequency are registered in COSMIC with CNT > 50.

Additional File 2 - Supplemental Figures

<u>ISOWN: accurate somatic mutation identification in the absence of normal tissue controls</u>

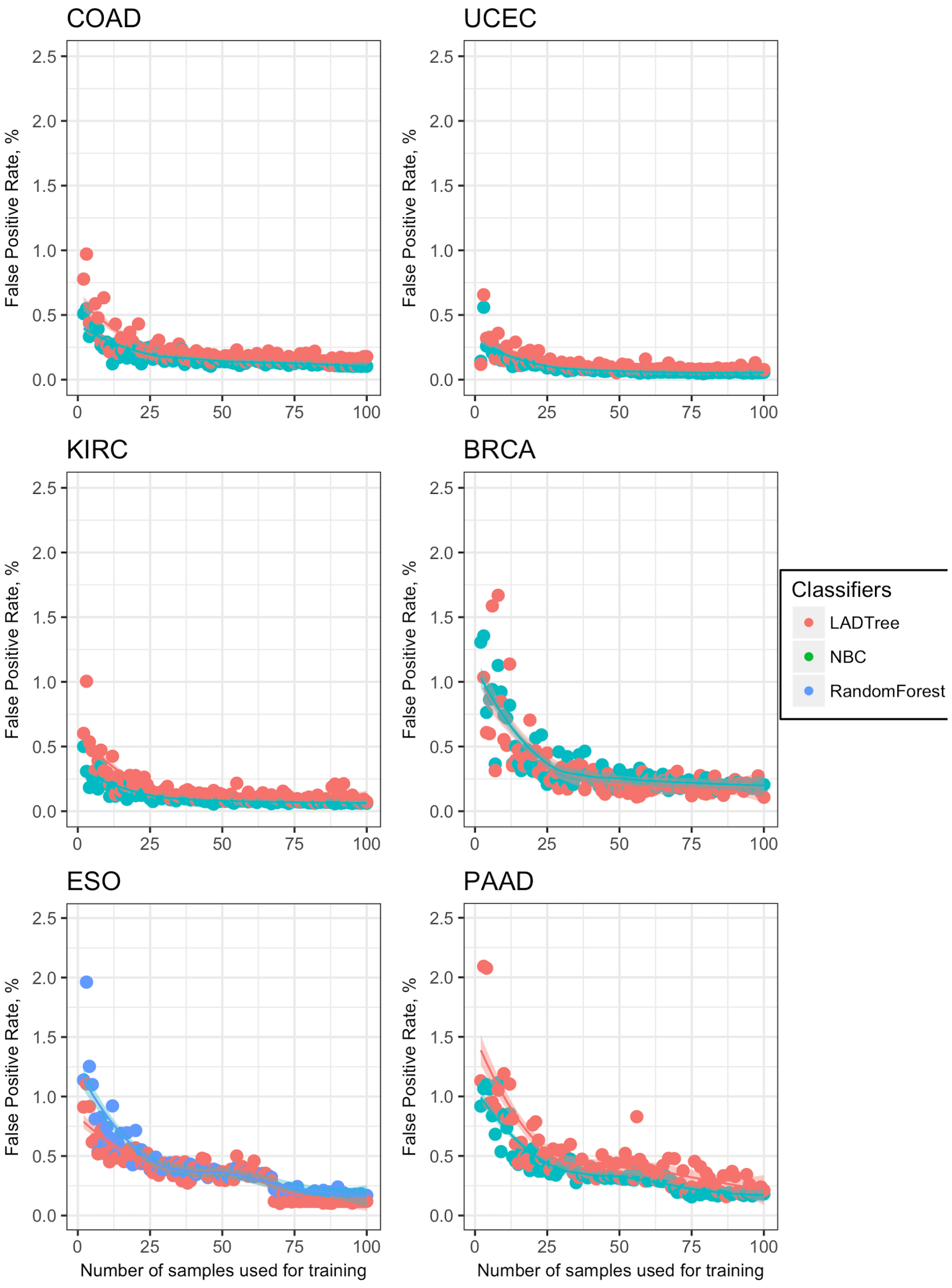

Suppl. Figure 5. ISOWN testing using two different machine learning algorithms in six whole-exome sequencing datasets. NBC (green), LADTree (red) and Random Forest (blue) algorithms were used for ISOWN validation. Classifiers were trained based on the gradually increased number of samples (indicated at x axes). False Positive Rate calculated based on held-out independent sample set across six cancer datasets.

Additional File 2 - Supplemental Figures

ISOWN: accurate somatic mutation identification in the absence of normal tissue control

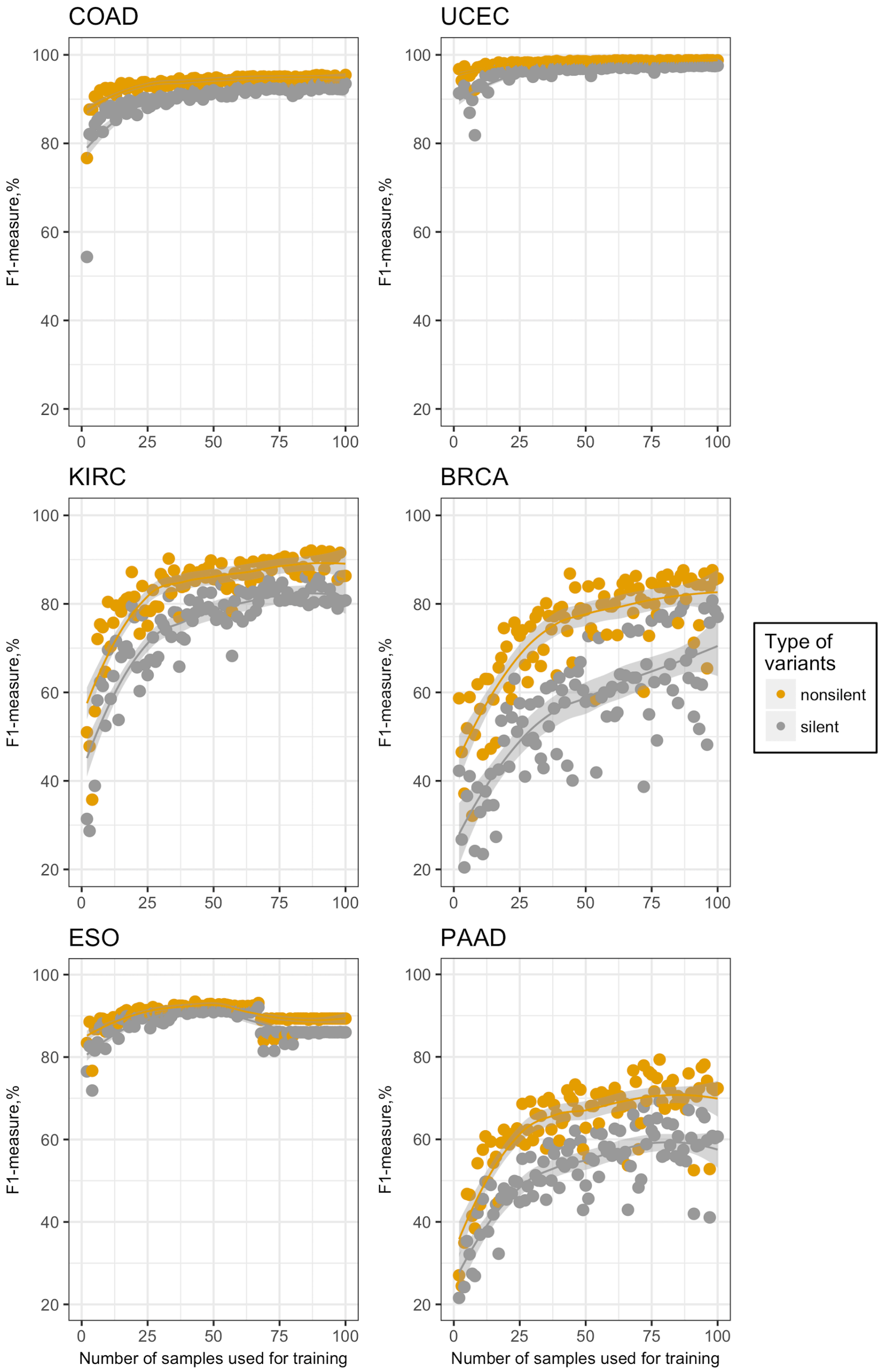

Suppl. Figure 6. ISOWN testing on different types of variants (silent vs non-silent variants). F1-measure calculated based on held-out independent sample set for six cancer datasets. LADTree was trained based on variants retrieved from the gradually increasing number of samples (indicated at x axes). Validation was done on either only non-silent (dark yellow plots) or only silent variants (grey plots).

Additional File 2 - Supplemental Figures

ISOWN: accurate somatic mutation identification in the absence of normal tissue controls

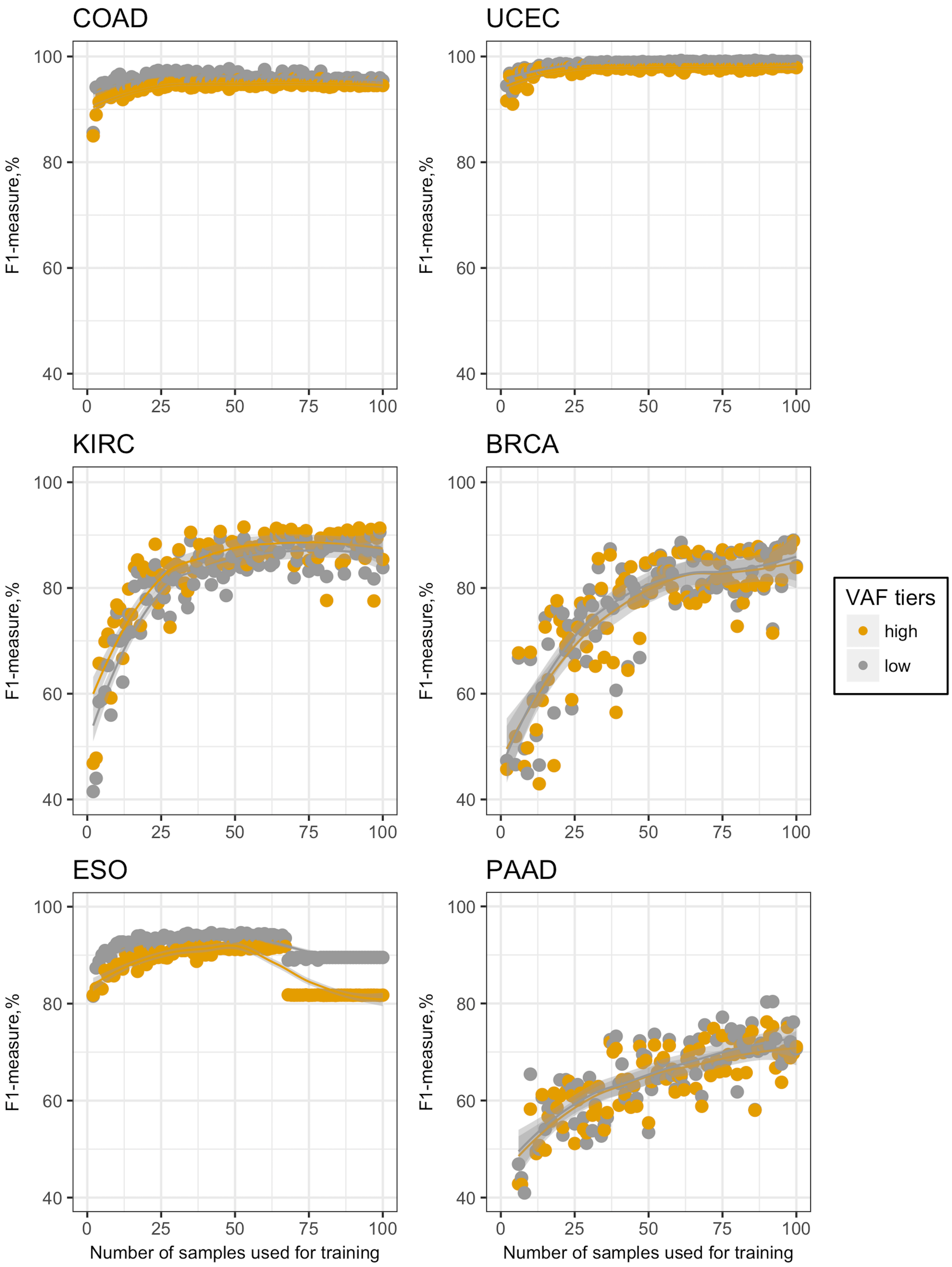

Suppl. Figure 7. ISOWN testing on different VAF tiers. F1-measure calculated based on held-out independent sample set for six cancer datasets. Classifier (LADTree) was trained based on the gradually increased number of samples (indicated at x axes). Validation was done on either low-tier somatic mutations (grey plots) or high-tier VAF somatic mutations (dark yellow plots).

Additional File 2 - Supplemental Figures

ISOWN: accurate somatic mutation identification in the absence of normal tissue control

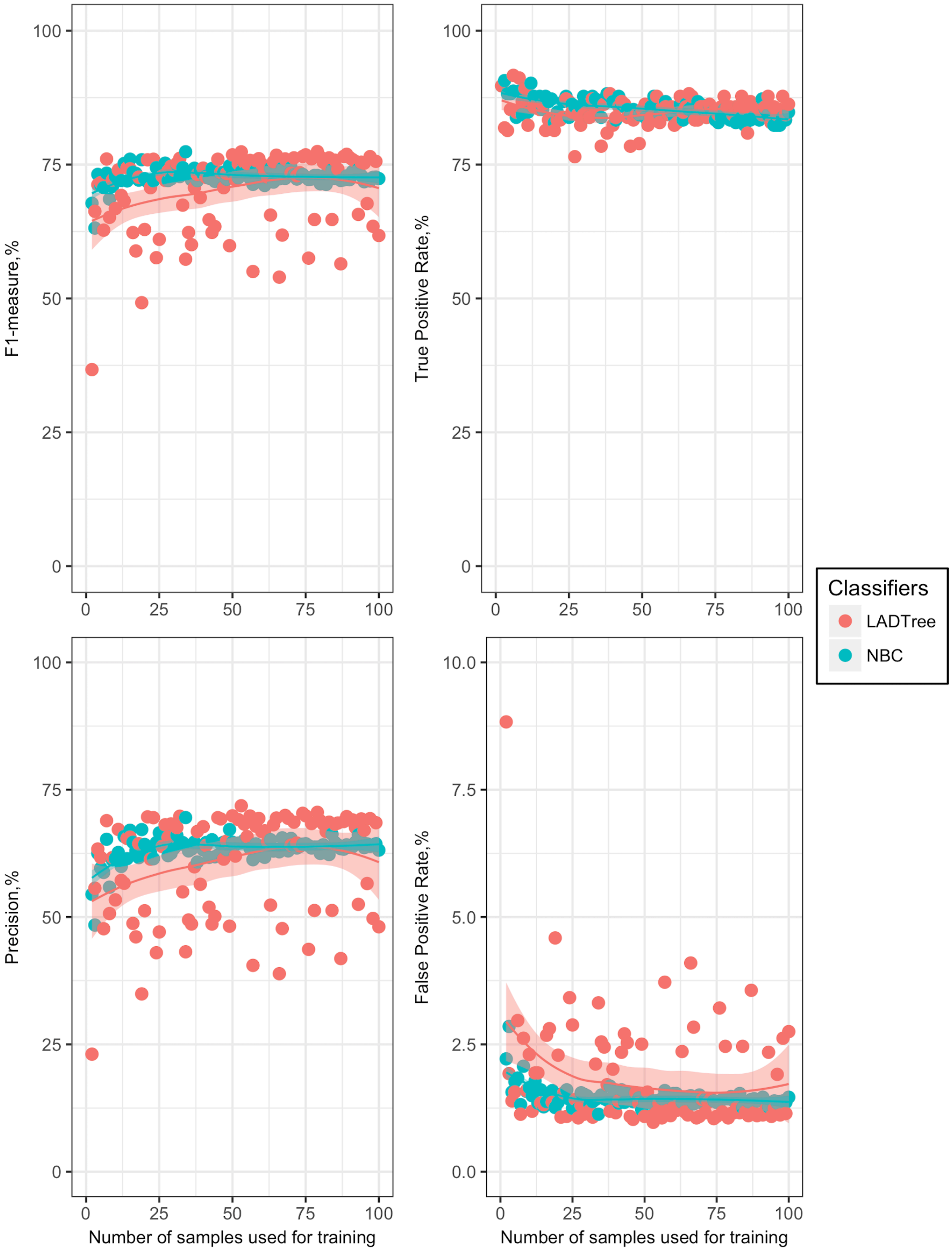

Suppl. Figure 8. ISOWN validation on cell lines. NBC was trained using a training set generated based on gradually increasing number of BRCA samples. Somatic mutations from 2 breast cancer cell lines (HCC1143 and HCC1954) were predicted. Sample frequency as a feature was removed from training and testing sets.
